# Mechanistic Insights into the Neurotoxicity of 2,5-Dimethoxyphenethylamines (2C) and Corresponding *N*-(2-methoxybenzyl)phenethylamine (NBOMe) Drugs

**DOI:** 10.3390/jox14020044

**Published:** 2024-06-05

**Authors:** Eva Gil-Martins, Fernando Cagide-Fagín, Daniel Martins, Ana Borer, Daniel José Barbosa, Carlos Fernandes, Daniel Chavarria, Fernando Remião, Fernanda Borges, Renata Silva

**Affiliations:** 1 Associate Laboratory i4HB-Institute for Health and Bioeconomy, Faculty of Pharmacy, University of Porto, 4050-313 Porto, Portugal; up201204736@edu.ff.up.pt (E.G.-M.); up201805228@edu.fc.up.pt (A.B.); remiao@ff.up.pt (F.R.); 2 UCIBIO-Applied Molecular Biosciences Unit, Laboratory of Toxicology, Department of Biological Sciences, Faculty of Pharmacy, University of Porto, 4050-313 Porto, Portugal; 3 CIQUP-IMS/Department of Chemistry and Biochemistry, Faculty of Sciences, University of Porto, 4169-007 Porto, Portugal; up200600201@edu.fc.up.pt (D.M.); carlos.fernandes@fc.up.pt (C.F.); daniel.chavarria@fc.up.pt (D.C.); fborges@fc.up.pt (F.B.); 4 Associate Laboratory i4HB-Institute for Health and Bioeconomy, University Institute of Health Sciences-CESPU, 4585-116 Gandra, Portugal; daniel.barbosa@iucs.cespu.pt; 5 UCIBIO-Applied Molecular Biosciences Unit, Translational Toxicology Research Laboratory, University Institute of Health Sciences (1H-TOXRUN, IUCS-CESPU), 4585-116 Gandra, Portugal; 6 i3S-Instituto de Investigação e Inovação em Saúde, Universidade do Porto, 4200-135 Porto, Portugal

**Keywords:** substituted phenethylamines, new psychoactive substances, 2C drugs, NBOMe drugs, neurotoxicity

## Abstract

Substituted phenethylamines including 2C (2,5-dimethoxyphenethylamines) and NBOMe (*N*-(2-methoxybenzyl)phenethylamines) drugs are potent psychoactive substances with little to no knowledge available on their toxicity. In the present in vitro study, we explored the mechanisms underlying the neurotoxicity of six substituted phenethylamines: 2C-T-2, 2C-T-4, 2C-T-7 and their corresponding NBOMes. These drugs were synthesized and chemically characterized, and their cytotoxicity (0–1000 μM) was evaluated in differentiated SH-SY5Y cells and primary rat cortical cultures, by the NR uptake and MTT reduction assays. In differentiated SH-SY5Y cells, mitochondrial membrane potential, intracellular ATP and calcium levels, reactive oxygen species production, and intracellular total glutathione levels were also evaluated. All the tested drugs exhibited concentration-dependent cytotoxic effects towards differentiated SH-SY5Y cells and primary rat cortical cultures. The NBOMe drugs presented higher cytotoxicity than their counterparts, which correlates with the drug’s lipophilicity. These cytotoxic effects were associated with mitochondrial dysfunction, evident through mitochondrial membrane depolarization and lowered intracellular ATP levels. Intracellular calcium imbalance was observed for 2C-T-7 and 25T7-NBOMe, implying a disrupted calcium regulation. Although reactive species levels remained unchanged, a reduction in intracellular total GSH content was observed. Overall, these findings contribute to a deeper understanding of these drugs, shedding light on the mechanisms underpinning their neurotoxicity.

## 1. Introduction

**Figure 1:**
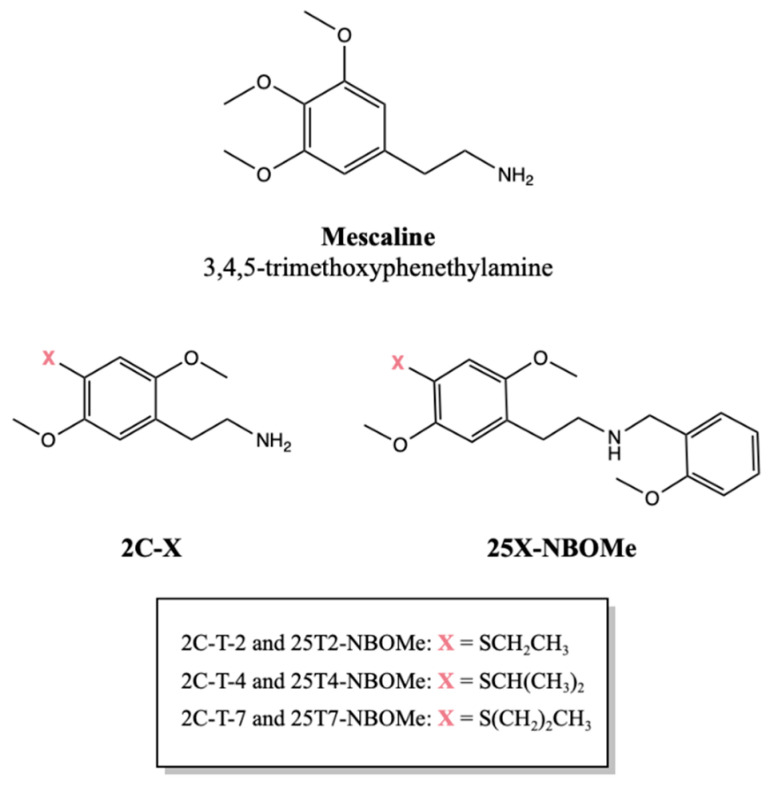
Chemical structures of mescaline, 2,5-dimethoxyphenethylamine (2C-X) drugs and their corresponding *N*-(2-methoxybenzyl)phenethylamine (25X-NBOMe) counterparts.

Psychedelic drugs are intricately woven into the fabric of human history. Over time, the use of natural psychedelics like psylocibin containing mushrooms and Ayahuasca brews progressed to include isolated compounds such as cocaine or morphine. As scientific methods evolved, entirely synthetic drugs like lysergic acid diethylamide (LSD) and methamphetamine appeared and gained popularity [1,2]. Many new synthetic drugs, also known as new psychoactive substances (NPS), are analogs or derivatives of naturally occurring drugs [3]. For instance, mescaline (Figure 1), which is present in several species of cactus (especially peyote cactus), served as a prototype during the 1970s for Alexander Shulgin to synthetize dozens of new synthetic drugs, including the 2C series (Figure 1). These drugs are ring-substituted phenethylamines characterized by the presence of two carbon atoms (2C) between the amine group and the aromatic ring [4]. Already in the 21st century, Ralf Heim, as part of his research into the pharmacology of serotonin receptors, synthesized the first NBOMe drugs by adding a 2-methoxybenzyl group to the amino group of the phenethylamine core [5] (Figure 1).

These drugs belong to the class of psychoactive substances that alter perception, mood, and cognition. Their psychedelic effects are mainly exerted through the activation of serotonin (5-HT) receptors, particularly the 5-HT_2A_ [6]. Moreover, the addition of the NBOMe moiety to the 2C drugs significantly enhances their binding affinity for 5-HT_2A_ receptors, creating remarkably potent 5-HT_2A_ receptor agonists [7].

Lately, an increasing number of reports highlighted the beneficial impact of psychedelic-based therapies on various neuropsychiatric disorders [8,9]. However, comparing to classic psychedelics (e.g., mescaline, LSD), these new drugs are more toxic, with several reports detailing severe adverse effects and even fatalities. The lack of regulation and oversight in their production and distribution often leads to unpredictable effects due to uncertain purity and contents, but users frequently manifest agitation, disorientation, visual and auditory hallucinations, tachycardia, and hypertension [10,11,12]. More serious clinical effects are usually associated with the NBOMes consumption and include rhabdomyolysis, metabolic acidosis, and multiorgan failure [13,14,15]. Beyond case reports, only a few studies explored the toxicological profile of these drugs, complicating the understanding of their potential impact on human health.

Thus, the main objective of this work was to collect information on the toxicological profile of a group of drugs from the 2C series—2C-T-2, 2C-T-4, and 2C-T-7—and their corresponding NBOMe counterparts—25T2-NBOMe, 25T4-NBOMe, and 25T7-NBOMe (Figure 1). The drugs were synthesized, and their in vitro neurotoxic profile was assessed using differentiated SH-SY5Y cells and primary rat cortical cultures. The putative pathways underlying the neurotoxicity induced by these drugs were also explored in differentiated SH-SY5Y cells, with particular focus on mitochondrial disfunction.

## 2. Materials and Methods

### 2.1. Chemistry

#### 2.1.1. Solvents, Reagents, and Experimental Conditions

All the organic solvents, reagents and materials used were of analytical grade, purchased from Carlo Erba Reagents (Emmendingen, Germany), Fluorochem (Glossop, United Kingdom), Sigma-Aldrich (Taufkirchen, Germany), TCI Chemicals (Tokyo, Japan), or Merck (Darmstadt, Germany). Thin-layer chromatography (TLC) was conducted on precoated silica gel sheet 60 F254 plates with a layer thickness of 0.2 mm (Merck, Darmstadt, Germany). Reaction progress was monitored using different proportions of ethyl acetate, dichloromethane, and dichloromethane/methanol. ^1^H and ^13^C Nuclear Magnetic Resonance (NMR) spectra of the samples were acquired at room temperature on a Bruker AMX 400 NMR spectrometer functioning at 400.14 MHz and 101.00 MHz, respectively. Tetramethylsilane (TMS) served as internal reference, chemical shifts were presented in ppm (δ), and coupling constants (J) provided in Hz. The physicochemical properties were determined on a reverse-phase NEXERA-i LC-2040C ultra-high-performance liquid chromatography (RP-UHPLC, Shimadzu, Kyoto, Japan) with an autosampler and a diode array detector (DAD). The RP-UHPLC chromatograms were obtained using a large bandwidth (190–800 nm), and the drugs retention times were extracted from the corresponding chromatograms using the Lab Solutions software (version 5.90 Shimadzu).

#### 2.1.2. Synthesis and Characterization of 2C-T-X and 25TX-NBOMe Drugs

The synthesis of 2,5-dimethoxybenzenesulfonic acid: Chlorosulfonic acid (15 mL) was added gradually over a solution of 1,4-dimethoxybenzene (8 g, 57.90 mmol) in DCM (50 mL). The resulting mixture was stirred for 1 h, and water (100 mL) was then added. After stirring for 10 min, the phases were separated, and the aqueous phase was extracted with DCM (3 × 75 mL). The organic phases were combined and dried over anhydrous Na_2_SO_4_. The organic solvent was evaporated under vacuum to obtain the desired product, which was used without further purification. Yield: 65%. ^1^H NMR (400 MHz, DMSO) δ 14.51 (1H, s, SO_2_OH), 7.28 (1H, d, J = 2.9 Hz, H6), 7.03–6.61 (2H, m, H3, H4), 3.72 (3H, s, OCH^3^), and 3.70 (3H, s, OCH^3^). ^13^C NMR (101 MHz, DMSO) δ 152.5 (C5), 150.8 (C2), 135.8 (C1), 116.1 (C6), 114.5 (C4), 114.2 (C3), 56.7 (OCH^3^), and 55.9 (OCH^3^).

The synthesis of 2,5-dimethoxybenzenethiol: Zinc powder (20 g, 305.0 mmol) was added to a solution of 2,5-dimethoxybenzenesulfonic acid (4 g, 18.5 mmol) in acetic acid (200 mL) and water (40 mL) under argon atmosphere. The suspension was stirred at 110 °C for 3 h. The solution was further diluted with water (1L), filtered to remove zinc residues, and extracted with DCM (3 × 75 mL). The organic layer was dried with anhydrous Na_2_SO_4_, filtered, and the solvent was evaporated yielding a yellow residue (Yield: 85%). ^1^H NMR (400 MHz, DMSO) δ 7.27 (1H, d, J = 2.9 Hz, H6), 6.95–6.86 (2H, m, H3 and H4), 3.71 (3H, s, OCH_3_), and 3.70 (3H, s, OCH_3_).

The synthesis of alkylsulfane derivatives: Potassium hydroxide (1.6 mmol) was added to 2,5-dimethoxybenzenethiol (1 mmol) dissolved in methanol (1.3 mL), and the suspension was stirred for 10 min. After that, the corresponding haloalkane was added and the reaction was stirred for 30–40 min. After methanol evaporation, the residue was dissolved in an aqueous solution of NaOH (0.5 M, 25 mL) and extracted with DCM (3 × 5 mL). The organic layer was dried with anhydrous Na_2_SO_4_, filtered, and evaporated.

(2,5-dimethoxyphenyl)(ethyl)sulfane. Yield: 82%. ^1^H NMR (400 MHz, DMSO) δ: 6.89 (1H, d, J = 8.8 Hz, H3), 6.76 (1H, d, J = 2.9 Hz, H6), 6.70 (1H, dd, J = 8.8, 3.0 Hz, H4), 3.74 (3H, s, OCH_3_), 3.71 (3H, s, OCH_3_), 2.89 (2H, q, J = 7.3 Hz, SCH_2_CH_3_), and 1.23 (3H, t, J = 7.3 Hz, SCH_2_CH_3_).
(2,5-dimethoxyphenyl)(isopropyl)sulfane. Yield: 89%. ^1^H NMR (400 MHz, DMSO) δ: 6.91 (1H, d, J = 8.9 Hz, H3), 6.84 (1H, d, J = 3.0 Hz, H6), 6.76 (1H, dd, J = 8.8, 3.0 Hz, H4), 3.73 (3H, s, OCH_3_), 3.71 (3H, s, OCH_3_), 3.51 (2H, hept, J = 6.7 Hz, SCH(CH_3_)_2_), and 1.22 (6H, d, J = 6.6 Hz, SCH(CH_3_)_2_). ^13^C NMR (101 MHz, DMSO) δ: 153.2 (C5), 151.4 (C2), 124.7 (C1), 116.1 (C3), 112.0 (C6), 111.4 (C4), 56.1 (OCH_3_), 55.3 (OCH_3_), 34.6 (SCH(CH_3_)_2_), and 22.6 (SCH(CH_3_)_2_).
(2,5-dimethoxyphenyl)(propyl)sulfane. Yield: 94%. ^1^H NMR (400 MHz, DMSO) δ: 6.88 (1H, d, J = 8.8 Hz, H3), 6.75 (1H, d, J = 2.9 Hz, H6), 6.70 (1H, dd, J = 8.8, 2.9 Hz, H4), 3.74 (3H, s, OCH_3_), 3.71 (3H, s, OCH_3_), 2.85 (2H, t, J = 7.2 Hz, SCH_2_CH_2_CH_3_), 1.59 (2H, h, J = 7.3 Hz, SCH_2_CH_2_CH_3_), and 0.98 (3H, t, J = 7.3 Hz, SCH_2_CH_2_CH_3_). ^13^C NMR (101 MHz, DMSO) δ: 153.6 (C5), 150.4 (C2), 126.2 (C1), 113.3 (C3), 111.7 (C6), 110.0 (C4), 56.1 (OCH_3_), 55.4 (OCH_3_), 32.2 (SCH_2_CH_2_CH_3_, 21.6 (SCH_2_CH_2_CH_3_), and 13.3 (SCH_2_CH_2_CH_3_).

The synthesis of *β*-nitrostyrene derivatives: Phosphorus oxychloride (2.3 mmol) was added over *N*-methylformanilide (2.5 mmol). The resulting mixture was stirred for 10 min, at 55 °C. Subsequently, the appropriated alkylsulfide (1 mmol) was added to the reactional medium. After stirring the mixture at 55 °C for 25 min, water (19 mL) was then added, and the mixture was stirred for 30 min at the same temperature. The solution was extracted with DCM (3 × 5 mL). The organic phase was dried with Na_2_SO_4_, filtered, and concentrated. The oil formed was dissolved in nitromethane (30 mmol) and ammonium acetate (0.2 mmol) was added. The reaction was refluxed for 2–7 h. Once the reaction was complete, the excess nitromethane was evaporated, and the solid formed was recrystallized from isopropanol to obtain the desired β-nitrostyrene.

(E)-(2,5-dimethoxy-4-(2-nitrovinyl)phenyl)(ethyl)sulfane. Yield: 88%. ^1^H NMR (400 MHz, CDCl_3_) δ: 8.12 (1H, d, J = 13.5 Hz, Hα), 7.83 (1H, d, J = 13.5 Hz, Hβ), 6.84 (1H, s, H6), 6.78 (1H, s, H3), 3.93 (3H, s, OCH_3_), 3.89 (3H, s, OCH_3_), 2.99 (2H, q, J = 7.4 Hz, SCH_2_CH_3_), and 1.40 (t, J = 7.4 Hz, SCH_2_CH_3_). ^13^C NMR (101 MHz, CDCl_3_) δ: 154.4 (C2), 150.4 (C5), 137.2 (Cβ), 135.2 (Cα), 134.3 (C4), 115.6 (C1), 112.3 (C3), 109.8 (C6), 56.5 (OCH_3_), 56.1 (OCH_3_), 25.4 (SCH_2_CH_3_), and 13.6 (SCH_2_CH_3_).
(E)-(2,5-dimethoxy-4-(2-nitrovinyl)phenyl)(isopropyl)sulfane. Yield: 52%. ^1^H NMR (400 MHz, DMSO) δ: 8.20 (2H, s, Hα, Hβ), 7.40 (1H, s, H6), 6.95 (1H, s, H3), 3.92 (3H, s, OCH_3_), 3.81 (3H, s, OCH_3_), 3.82–3.72 (1H, m, SCH(CH_3_)_2_), and 1.31 (6H, d, J = 6.6 Hz, SCH(CH_3_)_2_). ^13^C NMR (101 MHz, DMSO) δ: 153.7 (C2), 149.9 (C5), 136.8 (Cβ), 134.2 (C4), 133.6 (Cα), 114.9 (C1), 111.6 (C3), 110.7 (C6), 56.38 (OCH_3_), 56.37 (OCH_3_), 33.9 (SCH(CH_3_)_2_), and 22.4 (SCH(CH_3_)_2_).
(E)-(2,5-dimethoxy-4-(2-nitrovinyl)phenyl)(propyl)sulfane. Yield: 47%. ^1^H NMR (400 MHz, CDCl_3_) δ: 8.12 (1H, d, J = 13.5 Hz, Hα), 7.83 (1H, d, J = 13.5 Hz, Hβ), 6.83 (1H, s, H6), 6.77 (1H, s, H3), 3.93 (3H, s, OCH3), 3.89 (3H, s, OCH_3_), 2.94 (2H, t, J = 7.3 Hz, SCH_2_CH_2_CH_3_), 1.77 (2H, h, J = 7.4 Hz, SCH_2_CH_2_CH_3_), and 1.09 (3H, t, J = 7.4 Hz, SCH_2_CH_2_CH_3_). ^13^C NMR (101 MHz, CDCl_3_) δ: 154.4 (C2), 150.4 (C5), 137.2 (Cβ), 135.2 (Cα), 134.6 (C4), 115.5 (C1), 112.3 (C3), 109.7 (C6), 56.5 (OCH_3_), 56.1 (OCH_3_), 33.3 (SCH_2_CH_2_CH_3_), 22.0 (SCH_2_CH_2_CH_3_), and 13.7 (SCH_2_CH_2_CH_3_).

The synthesis of *β*-phenethylamines derivatives: Under argon, a solution of the β-nitrostyrene (1.0 mmol) in anhydrous tetrahydrofuran (THF, 15 mL) was added drop by drop to a stirred suspension of lithium aluminum hydride (LiAlH_4_, 9.0 mmol) in anhydrous THF (20 mL). The mixture was heated to reflux, stirred for 24 h, and after cooling to room temperature, the excess LiAlH_4_ was destroyed, at 0 °C, with the addition of isopropanol. Then, an aqueous solution of NaOH (4 M) was added to allow a complete conversion of aluminum salts to a loose and filterable solid residue. The suspension was then diluted with THF and the solid was filtered through celite. The organic solvent was removed by vacuum evaporation and the solution was acidified with HCl (40 mL). The aqueous solution was then washed with DCM (3 × 100 mL), basified with NaOH (4 M, pH ≈ 10–11), and extracted with DCM (3 × 100 mL). The organic layer was dried with anhydrous Na_2_SO_4_, filtered, and concentrated. The purification of the crude product was performed by flash chromatography in silica gel 60 (0.040–0.063 mm) using a methanol-DCM mixture (1:9 and 2:8) as mobile phase. The final oils were treated with 2 M HCl ethereal solution, obtaining the salts.

2-(4-(ethylthio)-2,5-dimethoxyphenyl)ethan-1-aminium chloride (2C-T-2). Yield: 46%. ^1^H NMR (400 MHz, MeOD) δ: 6.92 (1H, s, H6), 6.84 (1H, s, H3), 3.83 (3H, s, OCH_3_), 3.82 (3H, s, OCH_3_), 3.17–3.09 (2H, m, Hα), 2.98–2.86 (4H, m, Hβ and SCH_2_CH_3_), and 1.26 (3H, t, J = 7.4 Hz, 1H SCH_2_CH_3_). ^13^C NMR (101 MHz, MeOD) δ: 151.9 (C2 and C5), 124.5 (C1), 123.0 (C4), 113.9 (C6), 112.7 (C3), 55.7 (OCH_3_), 55.1 (OCH_3_), 39.5 (Cα), 28.3 (Cβ), 25.7 (SCH_2_CH_3_), and 13.2 (SCH_2_CH_3_).
2-(4-(isopropylthio)-2,5-dimethoxyphenyl)ethan-1-aminium chloride (2C-T-4). Yield: 59%. ^1^H NMR (400 MHz, MeOD) δ: 6.99 (1H, s, H6), 6.87 (1H, s, H3), 3.82 (6H, s, 2xOCH_3_), 3.50 (1H, hept, J = 6.7 Hz, SCH(CH_3_)_2_), 3.14 (2H, t, J = 7.4 Hz, Hα), 2.95 (2H, t, J = 7.4 Hz, Hβ), and 1.23 (6H, d, J = 6.7 Hz, SCH(CH_3_)_2_). ^13^C NMR (101 MHz, MeOD) δ: 154.6 (C2), 152.9 (C5), 125.8 (C1), 124.8 (C4), 117.2 (C6), 115.5 (C3), 57.1 (OCH_3_), 56.5 (OCH_3_), 40.9 (Cα), 37.6 (SCH(CH_3_)_2_), 29.8 (Cβ), and 23.4 (SCH(CH_3_)_2_).
2-(2,5-dimethoxy-4-(propylthio)phenyl)ethan-1-aminium chloride (2C-T-7). Yield: 66%. ^1^H NMR (400 MHz, MeOD) δ: 6.92 (1H, s, H6), 6.84 (1H, s, H3), 3.83 (3H, s, OCH_3_), 3.82 (s, OCH_3_), 3.13 (2H, t, J = 7.4 Hz, Hα), 2.94 (2H, t, J = 7.4 Hz, Hβ), 2.87 (2H, t, J = 7.2 Hz, SCH_2_CH_2_CH_3_), 1.63 (2H, h, J = 7.3 Hz, SCH_2_CH_2_CH_3_), and 1.02 (3H, t, J = 7.4 Hz, SCH_2_CH_2_CH_3_). ^13^C NMR (101 MHz, MeOD) δ: 153.4 (C2), 153.2 (C5), 126.1 (C1), 124.4 (C4), 115.3 (C6), 114.1 (C3), 57.1 (OCH_3_), 56.5 (OCH_3_), 40.9 (Cα), 35.2 (SCH_2_CH_2_CH_3_), 29.7 (Cβ), 23.5 (SCH_2_CH_2_CH_3_), and 13.7 (SCH_2_CH_2_CH_3_).

The synthesis of *N*-benzyl-β-phenethylamines derivatives: A solution of β-phenethylamine (1.0 mmol) and the analogous aldehyde (1.0 mmol) in 10 mL of ethanol was stirred for 5 h. After the formation of the imine intermediate NaBH_4_ (2.0 mmol) was added, and the reaction stirred for another hour. Under reduced pressure, the solvent was removed, and the residue was dissolved in ethyl acetate (20 mL) and washed with water (2 × 30 mL). The organic layer was dried with anhydrous Na_2_SO_4_, filtered, and evaporated. The crude oil was purified by flash chromatography in silica gel 60 (0.040–0.063 mm) using a methanol dichloromethane mixture (1:9 and 2:8) as the mobile phase. The final oils were treated with 2 M HCl ethereal solution, obtaining the salts. The salts were then recrystallized from ether.

2-(4-(ethylthio)-2,5-dimethoxyphenyl)-*N*-(2-methoxybenzyl)ethan-1-aminium chloride (25T2-NBOMe). Yield: 87%. ^1^H NMR (400 MHz, MeOD) δ: 7.46 (1H, ddd, J = 8.3, 7.4, 1.7 Hz, H4′), 7.37 (1H, dd, J = 7.5, 1.6 Hz, H6′), 7.09 (1H, d, J = 8.3 Hz, H3′), 7.02 (1H, ddd, J = 7.5, 7.5, 0.9 Hz, H5′), 6.91 (1H, s, H6), 6.84 (1H, s, H3), 4.24 (2H, s, NCH_2_Ph), 3.88 (3H, s, OCH_3_), 3.81 (3H, s, OCH_3_), 3.80 (3H, s, OCH_3_), 3.26–3.18 (2H, m, Hα), 3.07–2.96 (m, Hβ), 2.91 (q, J = 7.4 Hz, SCH_2_CH_3_), and 1.27 (3H, t, J = 7.4 Hz, SCH_2_CH_3_). ^13^C NMR (101 MHz, MeOD) δ: 159.4 (C2′), 153.3 (C2), 153.1 (C5), 132.8 (C6′), 132.7 (C4′), 126.3 (C1), 124.0 (C1′), 122.1 (C5′), 120.3 (C4), 115.2 (C3′), 114.0 (C6), 112.2 (C3), 57.1 (OCH_3_), 56.6 (OCH_3_), 56.2 (OCH_3_), 48.2 (NCH_2_Ph), 48.1 (Cα), 28.3 (Cβ), 27.1 (SCH_2_CH_3_), and 14.6 (SCH_2_CH_3_).
2-(4-(isopropylthio)-2,5-dimethoxyphenyl)-*N*-(2-methoxybenzyl)ethan-1-aminium chloride (25T4-NBOMe). Yield: 37%. ^1^H NMR (400 MHz, MeOD) δ: 7.46 (1H, ddd, J = 8.3, 7.5, 1.7 Hz, H4′), 7.37 (1H, dd, J = 7.5, 1.7 Hz, H6′), 7.09 (1H, dd, J = 8.3, 1.0 Hz, H3′), 7.02 (1H, ddd, J = 7.5 Hz, 1.0 Hz,, H5′), 6.99 (1H, s, H6), 6.86 (1H, s, H3), 4.24 (2H, s, NCH_2_Ph), 3.89 (3H, s, OCH_3_), 3.81 (3H, s, OCH_3_), 3.79 (3H, s, OCH_3_), 3.55–3.45 (2H, m, SCH(CH_3_)_2_), 3.27–3.18 (2H, m, Hα), 3.07–2.98 (2H, m, Hβ), and 1.23 (6H, d, J = 6.7 Hz, SCH(CH_3_)_2_).
2-(2,5-dimethoxy-4-(propylthio)phenyl)-N-(2-methoxybenzyl)ethan-1-aminium chloride (25T7-NBOMe). Yield: 84%. ^1^H NMR (400 MHz, MeOD) δ: 7.50–7.41 (1H, m, 1H4′), 7.37 (1H, dd, J = 7.5, 1.5 Hz, H6′), 7.09 (1H, d, J = 8.3 Hz, H3′), 7.02 (1H, dd, J = 7.5, 7.5 Hz, H5′), 6.91 (1H, s, H6), 6.84 (1H, s, H3), 4.24 (2H, s, NCH_2_Ph), 3.88 (3H, s, OCH3), 3.81 (3H, s, OCH_3_), 3.80 (3H, s, OCH_3_), 3.28–3.17 (1H, m, Hα), 3.05–2.91 (1H, m, Hβ), 2.87 (2H, t, J = 7.2 Hz, SCH_2_CH_2_CH_3_), 1.63 (2H, h, J = 7.3 Hz, SCH_2_CH_2_CH_3_), and 1.03 (3H, t, J = 7.4 Hz, SCH_2_CH_2_CH_3_). ^13^C NMR (101 MHz, MeOD) δ: 159.4 (C2′), 153.4 (C2), 153.1 (C5), 132.8 (C6′), 132.7 (C4′), 124.0 (C1′), 122.1 (C5′), 120.3 (C4), 115.2 (C2), 114.1 (C6), 112.2 (C3), 57.1 (OCH_3_), 56.6 (OCH_3_), 56.2 (OCH3), 48.2 (NCH_2_Ph), 48.1 (Cα), 35.2 (SCH_2_CH_2_CH_3_), 28.3 (Cβ), 23.6 (SCH_2_CH_2_CH_3_), and 13.7 (SCH_2_CH_2_CH_3_).

### 2.2. Determination of the Chromatographic Hydrophobicity Index (CHI)

The determination of the Chromatographic Hydrophobicity Index (CHI) values was carried out as described elsewhere [16].

CHI values were estimated from the retention times (tR) of the drugs and a blend of reference compounds acquired on a RP-UHPLC system with a Luna C18 (2) column (150 × 4.6 mm, 3 µm, Phenomenex, Torrance, LA, USA). To obtain a final concentration of 250 μM, the stock solutions of the drugs (10 mM, in DMSO), were diluted with an acetonitrile/water (1:1) mixture. The chromatographic analysis was firstly carried out at a physiological pH, but notable peak tailing, fronting, and splitting were observed and, as such, it is not reliable for accurate determinations. These types of chromatographic drawbacks can occur along the analysis of amines and in accordance; the analysis must occur at a pH value where only uncharged species are present. For this reason, the experiments were conducted at pH 2.3. Therefore, the mobile phase A was 1% aqueous acetic acid solution (pH = 2.3), and mobile phase B was acetonitrile. The following gradient program was applied: 0–7 min 0–100% B, 7–10 min 100% B, 10–12 min 100–0% B. The analytical system was operated at 35 °C. The flow rate was 1 mL/min, and the injection volume was 20 μL. A calibration line was obtained using a blend of the following reference compounds: theophylline, paracetamol, caffeine, benzimidazole, colchicine, carbamazepine, indole, propiophenone, butyrophenone, and valerophenone (Supplementary Figure S1) [17]. As previously described [16], the CHI LogD at pH 2.3 (CHILogD_2.3_) values were determined from the obtained CHI values using the following Equation (1):

CHILogD_2.3_ = 0.0525 CHI − 1.467 (1)

### 2.3. Chemicals and Materials for the In Vitro Studies

Dulbecco’s Modified Eagle’s Medium (DMEM)-high glucose, sodium bicarbonate, retinoic acid (RA), phorbol 12-myristate 13-acetate (TPA), neutral red solution (NR), thiazolyl blue tetrazolium bromide (MTT), tert-butyl hydroperoxide solution, 2′,7′-dichlorofluorescin diacetate (DCFH-DA), clorgyline, rasagiline mesylate, kynuramine, (R)-selegiline, L-buthionine sulfoximine (BSO), 5,5′-dithiobis(2-nitrobenzoic acid) (DTNB), glutathione reductase from baker’s yeast (*S. cerevisiae*), L-glutathione reduced (GSH), luciferase from *Photinus pyralis* (firefly), carbonyl cyanide 3-chlorophenylhydrazone (CCCP), and Poly-l-lysine (PLL) were obtained from Sigma-Aldrich (Taufkirchen, Germany). Microsomal MAO isoforms prepared from insect cells (BTI-TN-5B1-4) infected with recombinant baculovirus containing cDNA inserts for *h*MAO-A or *h*MAO-B were also purchased from Sigma-Aldrich (Taufkirchen, Germany). Neurobasal medium, B-27 supplement, GlutaMAX^TM^ supplement, heat inactivated horse serum, 0.5% trypsin-EDTA solution without phenol red, and Dulbecco’s phosphate-buffered saline (DPBS) were purchased from Gibco Laboratories (Waltham, MA, USA). DNAse recombinant I was obtained from Roche Diagnostics (Rotkreuz, Switzerland). Antibiotic cocktail (10,000 U/mL penicillin, 10,000 μg/mL streptomycin), heat-inactivated fetal bovine serum (FBS), non-essential amino acids solution, and Hanks’ balanced salt solution (HBSS) were obtained from PAN-Biotech (Aidenbach, Germany). β-nicotinamide adenine dinucleotide 2′-phosphate reduced tetrasodium salt (NADPH) was obtained from AppliChem (Darmstadt, Germany). DC™ protein assay kit was obtained from Bio-Rad (Hercules, CA, USA). Adenosine 5′-triphosphate (ATP) disodium salt hydrate, JC-1 dye, and Fluo-4 AM were obtained from Thermo Fisher Scientific (Waltham, MA, USA). D-luciferin potassium salt was obtained from Abcam (Cambridge, United Kingdom). Dimethyl sulfoxide (DMSO) was obtained from Merck (Darmstadt, Germany). All sterile plastic material was obtained from TPP (Trasadingen, Switzerland). All the reagents were of analytical grade or of the highest available grade.

Drug stock solutions were prepared in DMSO, stored at −20 °C, and freshly diluted on the day of the experiment in a complete cell culture medium.

### 2.4. Isolation of Primary Rat Cortical Neurons

Female Wistar rats were obtained from Charles River Laboratories. The animals were allowed free access to rat chow and water, being maintained on a 12 h/12 h light/dark cycle, under controlled temperature (20 ± 2 °C) and humidity (40–60%). Primary cortical cultures were prepared as previously described [18]. Briefly, for embryo collection, the pregnant female Wistar rats were anesthetized with isoflurane and subsequently administered ketamine and xylazine (200 + 100 μL, intraperitoneal). At the end of the dissection procedures, the female rat was euthanized with the administration of sodium pentobarbital (intracutaneous). The embryos were used between embryonic day 16 (E16) and E19, considering the mating day as E0. The embryos were dissected from the uterus, and the heads were decapitated and placed in ice-cold 0.1 M PBS supplemented with 0.6% (*w*/*v*) glucose. The cortex dissection was performed under a Zeiss stereo microscope (Stemi DV4). Following trypsinization (0.05% trypsin/EDTA) and DNAse I treatment, the tissue pieces were mechanically dissociated, and the cells seeded in freshly prepared neurobasal medium (supplemented with 2% B-27, 100 U/mL of penicillin, 100 μg/mL of streptomycin, and 1% GlutaMAX^TM^ supplement) at a density of 150,000 cells/cm^2^ in 96-well plates precoated, overnight, with 0.05 mg/mL PLL solution (prepared in 0.1 M borate buffer, pH 8.5). The cultures were maintained at 37 °C in a 5% CO_2_ air atmosphere. The medium was renewed at 4 days in vitro (DIV) by replacing half of the volume with fresh medium, and the cultures were used at 8 DIV.

This investigation followed the highest standards of ethics after approval by the local Ethical Committee for the Welfare of Experimental Animals (University of Porto-ORBEA) and by the national authority Direção-Geral de Alimentação e Veterinária (DGAV). Housing and experimental procedures were conducted by accredited investigators for laboratory animal use, aligning with the Portuguese and European legislation (law DL 113/2013, Guide for Animal Care; Directives 86/609/EEC and 2010/63/UE), under close the supervision of veterinary physicians. All procedures were taken to minimize the number of animals used and their suffering.

### 2.5. Cell Culture and Differentiation

The SH-SY5Y was acquired from the American Type Culture Collection (ATCC, Manassas, VA, USA).

SH-SY5Y cells were cultured in 25 cm^2^ flasks using DMEM-high glucose medium, supplemented with 10% fetal bovine serum (FBS), 1% nonessential amino acids (100 μM), and 1% antibiotic (10,000 U/mL penicillin and 10,000 μg/mL streptomycin). The cells were sustained in a 5% CO_2_-95% air atmosphere, at 37 °C.

The cells were seeded at a density of 25,000 cells/cm^2^. To achieve a more mature neuron-like phenotype, SH-SY5Y cells were initially cultured in a complete medium supplemented with RA (10 µM). Subsequently, 3 days post-seeding, TPA (80 nM) was added to each well, and the cells were maintained for another 3 days until use, thus achieving a dopaminergic phenotype [19]. In all the experiments, the cells were used between passages 22 and 28.

### 2.6. Evaluation of the Phenethylamine Derivatives Cytotoxicity

The differentiated SH-SY5Y cells (25,000 cells/cm^2^) and primary rat cortical cultures (150,000 cells/cm^2^) were seeded onto 96-well plates as previously described.

Accordingly, 6 days after seeding (differentiated SH-SY5Y cells) or at 8 DIV (primary cultures), the cells were exposed, for 24 h (37 °C, 5% CO_2_), to different concentrations of the drugs (0–1000 µM). Thus, the tested concentrations ranged from 0 to 1000 µM for 2C-T-2 and 2C-T-4; 0 to 500 µM for 2C-T-7; 0 to 250 µM for 25T2-NBOMe, and 0 to 100 µM for 25T4-NBOMe and 25T7-NBOMe.

At the end of the incubation period, the cell viability was assessed by the NR uptake and the MTT reduction assays. For primary rat cortical cultures, only the NR uptake assay was used.

#### 2.6.1. Neutral Red (NR) Uptake Assay

The NR uptake assay is based on the detection of viable cells through the incorporation of the NR dye by the cell’s lysosomes [20]. In brief, after 24 h of exposure to the tested drugs, the differentiated SH-SY5Y cells and primary rat cortical cultures were incubated with NR (50 µg/mL in complete medium) for 90 or 210 min, respectively, at 37 °C, in a 5% CO_2_-95% air atmosphere. To extract the absorbed dye, an acidified ethanol solution was used, and the absorbance was then measured at 540 nm in a multi-well plate reader (PowerWaveX, BioTek Instruments, Winooski, VT, USA) [21]. The cytotoxicity was estimated by the percentage of NR uptake relative to the control cells, and a minimum of 4 independent experiments were conducted in triplicate.

#### 2.6.2. MTT Reduction Assay

The MTT reduction assay is based on the capacity of metabolically active cells to reduce the water-soluble yellow tetrazolium dye (MTT) into water-insoluble formazan crystals [22]. In brief, after 24 h of exposure to the tested drugs, the differentiated SH-SY5Y cells were incubated with MTT (0.5 mg/mL in complete medium) for 90 min, at 37 °C, in a 5% CO_2_-95% air atmosphere. To solubilize the formed formazan crystals, an organic solvent (DMSO) was used, and the absorbance was then measured at 550 nm in a multi-well plate reader (PowerWaveX, BioTek Instruments, Winooski, VT, USA) [21]. The cytotoxicity was estimated by the percentage of MTT reduction relative to the control cells, and a minimum of 4 independent experiments were conducted in triplicate.

### 2.7. Evaluation of Mitochondrial Integrity through the JC-1 Dye

Mitochondrial membrane potential (ΔΨm) is a valuable parameter that reflects the energy status and functional integrity of cells, and the JC-1 dye is widely used as an indicator of ΔΨm. In healthy cells with high ΔΨm, JC-1 forms J-aggregates that emit red fluorescence. In apoptotic or unhealthy cells with low ΔΨm, JC-1 remains in its monomeric form and emits green fluorescence. The ratio of red to green fluorescence intensity is proportional to the degree of polarization of the mitochondrial membrane (a higher ratio indicates a more polarized membrane) [23].

Thus, the SH-SY5Y cells were seeded onto 24-well plates (25,000 cells/cm^2^) following the previously described seeding and differentiation protocol. On the day of the experiment, the cells were exposed to different concentrations of the tested drugs (EC_20_ and EC_50_) for 24 h (37 °C, 5% CO_2_). CCCP (100 µM, 2 h) was used as a positive control for mitochondrial membrane depolarization. At the end of the incubation period, the medium was removed, and the cells were incubated, for 30 min, with 10 µM JC-1 under light protection (37 °C, 5% CO_2_). Subsequently, the plates were centrifuged (5 min, 300× *g*, room temperature), the supernatants were rejected, and the cells were washed 2 times with warm HBSS with calcium and magnesium. Fluorescence was recorded at the wavelengths of 485 nm excitation and 535 nm emission for JC-1 monomers and 535 nm excitation and 595 nm emission wavelengths for J-aggregates in a multi-well plate reader (PowerWaveX, BioTek Instruments, Winooski, VT, USA). The results are presented as the percentage of JC-1 red/green fluorescence ratio relative to the control cells, and a minimum of 5 independent experiments were conducted in triplicate.

### 2.8. Quantification of the Intracellular Adenosine Triphosphate (ATP) Levels

Adenosine triphosphate (ATP) is often considered the primary energy currency of cells, being essential for several cellular activities, such as active transport, metabolism, and cell signaling, among many others [24]. The intracellular ATP levels were measured using a bioluminescence method, consisting of the reaction between luciferin and ATP in the presence of luciferase, where the resulting light intensity is directly proportional to the amount of ATP present in the cells [25].

Thus, the SH-SY5Y cells were seeded onto 6-well plates (25,000 cells/cm^2^) following the previously described seeding and differentiation protocol. On the day of the experiment, the cells were exposed to different concentrations of the tested drugs (EC_20_ and EC_50_) for 24 h (37 °C, 5% CO_2_). At the end of the incubation period, the cells were centrifuged (5 min, 250× *g*, 4 °C), the supernatants were rejected, and proteins were precipitated with 5% HClO_4_ for at least 30 min (4 °C). Subsequently, the acidic samples were collected from the plates, centrifuged (5 min, 16,000× *g*, 4 °C), and the supernatants were stored until further ATP analysis (−80 °C). To assess protein content, the pellets were resuspended in 1 M NaOH, maintained at 4 °C overnight, and stored until further analysis (−20 °C). A Bio-Rad DC™ protein assay kit (Bio-Rad Laboratories, Hercules, CA, USA) was used to analyze protein content, in accordance with the manufacturer’s guidelines.

Before the ATP quantification, the acidic samples were diluted (1:5) with 5% HClO_4_, and the diluted samples/standards were neutralized with an ice-cold KHCO_3_ solution (1:1) and centrifuged (16,000× *g*, 5 min, 4 °C). Then, 75 µL of each sample/standard supernatant was transferred into a white 96-well plate, 75 µL of a luciferin (300 µM)-luciferase (3,000,000 U/mL) solution (10–90%, respectively) was added, and bioluminescence (560 nm, 28 °C) was immediately measured in a multi-well plate reader (PowerWaveX, BioTek Instruments, Winooski, VT, USA).

ATP levels were standardized to protein content. The results are presented as a percentage relative to the control cells (0 µM). A minimum of 6 independent experiments were conducted in duplicate.

### 2.9. Quantification of Intracellular Calcium Levels through the Fluo-4 AM Probe

Calcium is a vital second messenger for almost every cellular process, including cellular signaling, muscle function, neurotransmitter release, membrane function, and apoptosis. Therefore, its levels inside cells are strictly controlled to maintain cellular homeostasis [26]. The intracellular calcium levels were measured using the cell-permeable Fluo-4 AM probe. Inside the cells, Fluo-4 AM is cleaved to its active and fluorescent form—Fluo-4—which has a high affinity for calcium ions (Ca^2^⁺). Thus, its fluorescence emission is directly proportional to the concentration of calcium ions in the intracellular environment [27].

Therefore, the SH-SY5Y cells were seeded onto 96-well plates (25,000 cells/cm^2^) following the previously described seeding and differentiation protocol. On the day of the experiment, the cells were pre-incubated for 30 min with 3 µM Fluo-4 AM (protected from light, 37 °C, 5% CO_2_) and subsequently exposed to the drugs (EC_20_ and EC_50_) or negative/positive controls (500 µM of EGTA and 25 mM CaCl_2_, respectively), and the absorbance was immediately recorded, every 18 s, for 1 h (kinetic fluorescence mode), at an excitation wavelength of 488 nm and emission wavelength of 520 nm in a multi-well plate reader (PowerWaveX, BioTek Instruments, Winooski, VT, USA). The average value for negative control (EGTA-treated cells) was subtracted from every other condition and then the minimum average value was subtracted from the maximum average value for every other condition. The results are presented as a percentage relative to the control cells (0 µM). A minimum of 5 independent experiments were conducted in triplicate.

### 2.10. Quantification of the Intracellular Levels of Reactive Species through the DCFH-DA Probe

Reactive species play important roles in cell signaling and immune response. However, when their levels are excessive, they can cause oxidative stress and damage the cells [28]. The intracellular reactive oxygen species (ROS) and reactive nitrogen species (RNS) levels were measured using the cell-permeable DCFH-DA probe. Inside the cells, DCFH-DA is cleaved by cellular esterases originating DCFH, which is oxidized by ROS/RNS, resulting in the fluorescent 2′,7′-dichlorofluorescein (DCF). The intensity of DCF fluorescence is an indicator of the ROS/RNS levels in the cells, with a higher fluorescence intensity indicating higher ROS/RNS levels, and a lower intensity suggesting lower ROS/RNS levels [29].

Therefore, the SH-SY5Y cells were seeded onto 96-well plates (25,000 cells/cm^2^) following the previously described seeding and differentiation protocol. On the day of the experiment, the cells were pre-incubated for 1 h with 10 µM DCFH-DA (protected from light, 37 °C, 5% CO_2_) and subsequently exposed to the tested drugs (EC_20_, EC_50_ and EC_80_) for 24 h (37 °C, 5% CO_2_). Tert-butyl hyoperoxide (*t*-BHP, 100 µM, 24 h) was used as a positive control.

At the end of the incubation period, the DCF fluorescence was measured at an excitation wavelength of 485 nm and an emission wavelength of 590 nm in a multi-well plate reader (PowerWaveX, BioTek Instruments, Winooski, VT, USA). The detection of ROS/RNS was assessed by the percent of probe oxidation relative to the control cells (0 µM), and a minimum of 4 independent experiments were conducted in triplicate.

### 2.11. Quantification of the Intracellular Total Glutathione (tGSH) Levels

Glutathione has a critical role in cellular physiology, playing multiple essential roles in maintaining cellular health and function, including antioxidant defense, detoxification, and cellular signaling [30]. The 5,5′-dithiobis(2-nitrobenzoic acid) (DTNB)-GSH recycling assay was used to measure total GSH (tGSH), which includes the reduced (GSH) and oxidized (GSSG) forms of glutathione. The assay relies on the reaction of GSH with DTNB, producing the yellow-colored 5-thio-2-nitrobenzoic acid (TNB). Thus, the TNB generated is directly proportional to the amount of GSH present in the sample [31].

Thus, the SH-SY5Y cells were seeded onto 6-well plates (25,000 cells/cm^2^) following the previously described seeding and differentiation protocol. On the day of the experiment, the cells were exposed to different concentrations of the tested drugs (EC_20_ and EC_50_) for 24 h (37 °C, 5% CO_2_). At the end of the incubation period, the cells were centrifuged (5 min, 250 g, 4 °C). For the analysis of extracellular GSH levels, the supernatants were collected, and proteins precipitated with 30% HClO_4_ for at least 30 min (4 °C). For the analysis of intracellular GSH levels, after collecting the supernatants, the cells were treated with 5% HClO_4_ for at least 30 min (4 °C). Subsequently, the acidic samples were collected from the plates and centrifuged (5 min, 16,000× *g*, 4 °C), and the supernatants were stored until further analysis (−80 °C). To assess protein content, the pellets were resuspended in 1M NaOH, maintained at 4 °C overnight, and stored until further analysis (−20 °C). A Bio-Rad DC™ protein assay kit (Bio-Rad Laboratories, Hercules, CA, USA) was used to analyze protein content, in accordance with the manufacturer’s guidelines.

Before the tGSH quantification, the acidic samples were diluted (1:2) with 5% HClO_4_, and the diluted samples/standards were neutralized with an ice-cold KHCO_3_ solution (1:1) and centrifuged (5 min, 16,000× *g*, 4 °C).

Then, 100 µL of supernatant from each sample/standard was transferred into a 96-well plate and 65 µL of an extemporaneous reagent solution (0.68 mM NADPH and 3.96 mM DTNB prepared in a 7.5 pH phosphate buffer) was added to each well, and the plates were incubated for 15 min at 30 °C. After 15 min, 40 μL of a 10 U/mL glutathione reductase solution was added to each well and the formation of 5-thio-2-nitrobenzoic acid (TNB) was monitored every 10 s for 3 min (kinetic mode) at 415 nm in a multi-well plate reader (PowerWaveX, BioTek Instruments, Winooski, VT, USA).

The GSH levels were standardized to protein content. The results are presented as a percentage relative to the control cells (0 µM). A minimum of 6 independent experiments were conducted, in duplicate.

### 2.12. Impact of Glutamate-Cysteine Ligase Inhibition on Drug-Induced Cytotoxicity

Glutamate/cysteine ligase (GCL), also recognized as gamma-glutamylcysteine synthetase (γ-GCS), is a key enzyme in the first step of glutathione biosynthesis, as it catalyzes the reaction between glutamate and cysteine to produce γ-glutamylcysteine [32]. Thus, the capacity of L-buthionine sulfoximine (BSO), an inhibitor of γ-GCS [33], to effectively inhibit this first rate-limiting enzyme of GSH biosynthesis under the intended experimental conditions (100 µM BSO for 24 h) was evaluated. The SH-SY5Y cells were seeded onto 6-well plates (25,000 cells/cm^2^) following the previously described seeding and differentiation protocol. On the day of the experiment, the cells were incubated with BSO (100 µM) for 24 h (37 °C, 5% CO_2_) and the quantification of GSH intracellular levels was achieved according to the previously described method in Section 2.11.

After proving the GSH depleting capacity of BSO, its impact on drug-induced cytotoxicity was assessed, with the aim of gaining a deeper understanding of GSH’s role in these drugs’ detoxification. Therefore, the SH-SY5Y cells were seeded onto 96-well plates (25,000 cells/cm^2^) following the previously described seeding and differentiation protocol. Accordingly, 5 days after seeding, the cells were pre-incubated for 24 h (37 °C, 5% CO_2_) with 100 µM BSO and subsequently exposed (6 days after seeding) to the tested drugs (EC_50_ and EC_80_) for 24 h (37 °C, 5% CO_2_). At the end of the incubation period, the drugs’ cytotoxicity was assessed through the NR uptake assay. A minimum of 4 independent experiments were conducted, in triplicate.

### 2.13. Impact of Monoamine Oxidases Inhibition on Drug-Induced Cytotoxicity

Monoamine oxidases (MAO) are essential enzymes in the regulation and metabolism of neurotransmitters, being also important in drug metabolism [34]. Thus, the SH-SY5Y cells (25,000 cells/cm^2^) were seeded and differentiated onto 96-well plates as previously described. On the experimental day, the cells were exposed to the tested drugs (EC_50_ and EC_80_) in the absence or presence of MAO inhibitors: clorgyline (1 μM, MAO-A inhibitor) or rasagiline (1 μM, MAO-B inhibitor). The cells were pre-exposed to MAO inhibitors for 1 h (37 °C, 5% CO_2_) before the tested drugs. After 24 h of exposure, cytotoxicity was assessed through the NR uptake assay. At least 4 independent experiments were performed in triplicate.

### 2.14. Assessment of Human Monoamine Oxidase (hMAO) Inhibitory Activity

The inhibitory activity of phenethylamine derivatives on *h*MAOs was studied using an experimental protocol described elsewhere [35,36]. The *h*MAO-A and *h*MAO-B inhibitory activities were evaluated in microsomal isoforms, prepared from insect cells (BTI-TN-5B1-4) infected with recombinant baculovirus carrying cDNA inserts for each MAO isoform, by quantifying the enzymatic transformation of kynuramine into 4-hydroxyquinoline. To ensure the same maximum velocity (V_max_ = 50 pmol.min^−1^) for each isoform—*h*MAO-A: 3 ng·µL^−1^ and *h*MAO-B: 12 ng·µL^−1^—the amounts of *h*MAO-A and *h*MAO-B were adjusted in our experimental conditions. All the assays were executed in 50 mM sodium phosphate-buffered solution at pH 7.4.

The drugs or reference inhibitors (final concentration: 10 µM) were pre-exposed at 37 °C for 10 min with kynuramine (K_m_ hMAO-A = 20 µM; K_m_ hMAO-B = 20 µM; final concentration: 2 × K_m_) in 96-well microplates (BRANDplates, pureGrade^TM^, BRAND GMBH, Germany). The reaction started with the addition of *h*MAO-A or *h*MAO-B. The spectrophotometric analysis of initial velocities was performed using a microplate reader (Synergy HT, BioTek Instruments, Winooski, VT, USA) at 37 °C by quantifying the formation of 4-hydroxyquinoline at 316 nm, over a minimum of 30 min (1 min intervals). The initial velocities, derived from the linear phase of product formation, were standardized to the control, yielding the percentages of the inhibition of the drugs (10 µM). The percentages of the MAO inhibition of each compound were determined from 3 independent experiments performed in triplicate.

### 2.15. Statistical Analysis

All the statistical calculations were performed using GraphPad Prism 9 for Windows (GraphPad Software, San Diego, CA, USA). Concentration/response (cytotoxicity) curves were achieved using the least squares as the fitting method and comparisons between curves (LOGEC_50_, TOP, BOTTOM, and Hill Slope) were made using the extra sum-of-squares F test. The normality of the data distribution was estimated by 3 tests (Kolmogorov/Smirnov, Shapiro/Wilk normality, and D’Agostino and Pearson omnibus tests). In experiments with only one variable, statistical comparisons between groups were performed with one-way ANOVA, followed by Dunnett’s or Tukey’s multiple comparisons *post hoc* test. Two-way ANOVA followed by Tukey’s multiple comparison *post hoc* test was applied to compare the data from experiments with two variables. The details of the statistical analysis performed are described in each figure legend. Differences were considered significant at *p* values below 0.05 and the results are presented as mean with 95% confidence interval (CI). For the primary cultures of rat cortical neurons, each independent experiment involved cortex cultures derived from different pregnant mice.

## 3. Results

**Figure 2:**
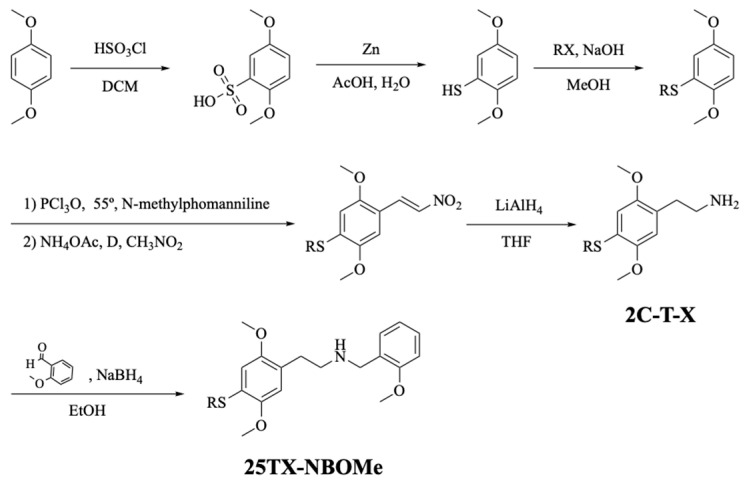
Synthetic route followed to obtain the 2C-T-X and 25TX-NBOMe drugs.

The drugs 2C-T-X and 25TX-NBOMe were synthesized following the strategy presented in Figure 2. First, 1,4-dimethoxybenzene was reacted with chlorosulfonic acid in dichloromethane (DCM) to obtain 2,5-dimethoxybenzenesulfonic acid. This compound was then reduced with zinc in acetic acid-water. At that point, different haloalkanes were added to the resulting sulfide in a basic medium, and the corresponding alkylsulfides were obtained. Then, an aldehyde group was introduced to each alkylsulfide by using *N*-methylformanilide, which was previously activated with phosphorus oxychloride. The aldehyde derivatives were reacted, under reflux, with nitromethane yielding the desired nitrostyrenes. The reduction of these nitrostyrenes with lithium aluminum hydride afforded 2C-T-X drugs. All the drugs were obtained in the form of hydrochloride salts to increase their solubility and stability. 25TX-NBOMe drugs were prepared from each 2C-T-X neutral base by a reductive amination reaction with sodium borohydride in ethanol. 25TX-NBOMe drugs were also obtained in the form of hydrochloride salts.

### 3.1. 2C-T-X and 25TX-NBOMe Drugs Induce Cell Death in a Concentration-Dependent Manner in Differentiated SH-SY5Y Cells and Primary Rat Cortical Neurons

**Figure 3:**
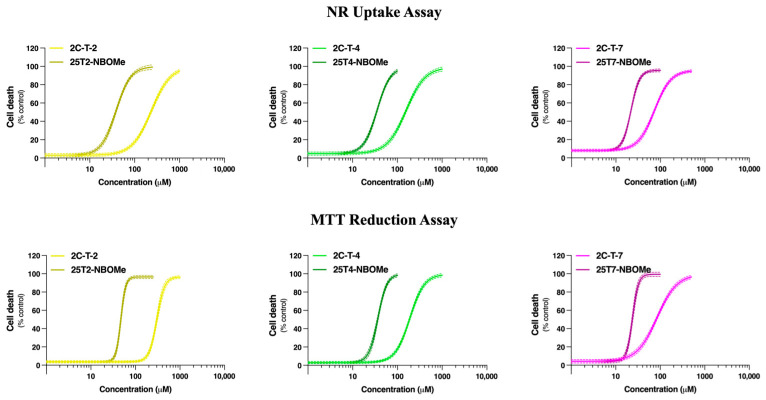
Concentration/response curves of the 2C-T-X (0–1000 µM) and 25TX-NBOMe drugs (0–250 µM) in the differentiated SH-SY5Y cells evaluated by the NR uptake and the MTT reduction assays, following 24 h of incubation. The concentration/response curves were fitted applying the least squares method and the results are presented as mean with a 95% confidence interval (CI) of a minimum of 4 different experiments (3 replicates each).

**Figure 4:**
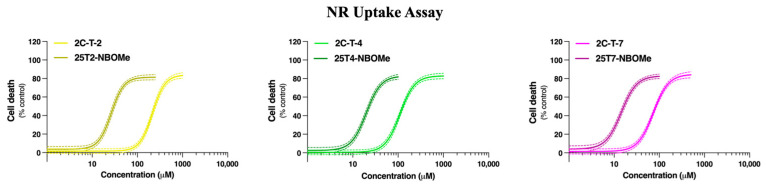
Concentration/response curves of the 2C-T-X and 25TX-NBOMe drugs (0–1000 µM) in the primary rat cortical cultures evaluated by the NR uptake assay, following 24 h of incubation. The concentration/response curves were fitted applying the least squares method and the results are presented as mean with a 95% confidence interval (CI) of 4 different experiments (3 replicates each).

The differentiated SH-SY5Y cells and primary rat cortical neurons were incubated for 24 h with growing concentrations of 2C-T-2 (0–1000 µM), 25T2-NBOMe (0–250 µM), 2C-T-4 (0–1000 µM), 25T4-NBOMe (0–100 µM), 2C-T-7 (0–500 µM), and 25T7-NBOMe (0–100 µM). Cytotoxicity was evaluated through the NR uptake (in the SH-SY5Y cells and in the primary rat cortical cultures) and MTT reduction (in the SH-SY5Y cells) assays. In the SH-SY5Y cells, for both assays, all the drugs caused a concentration-dependent cytotoxic effect, with the 25TX-NBOMe drugs presenting significantly higher cytotoxicity when compared to the 2C-T-X drugs, evidenced by the significant deviation of the concentration/response curve to the left (Figure 3), and by the significantly lower EC_50_ values for both cytotoxicity assays (Table 1). The NR uptake assay was more sensitive than the MTT reduction assay in the SH-SY5Y model, as observed by lower EC_50_ values for all the drugs (Table 1). Therefore, only the NR uptake assay was used to plot the concentration/response curves in the primary rat cortical cultures (Figure 4), which were similar but slightly more sensitive than the SH-SY5Y cells (Table 1).

For both cellular models and considering the NR uptake assay, the least cytotoxic pair of drugs was the 2C-T-2/25T2-NBOMe presenting EC_50_ values above 215 µM and 25 µM, respectively, while the most cytotoxic pair of drugs was the 2C-T-7/25T7-NBOMe, presenting EC_50_ values below 75 µM and 22 µM, respectively (Table 1).

Additionally, apart from the EC_50_ values, no significant differences were perceived in the comparison of other individual parameters of the fitted curves, namely Top, and Bottom for both models (Supplementary Table S1). Overall, the tested drugs showed similar cytotoxic profiles in the differentiated SH-SY5Y cells and primary rat cortical cultures. Thus, the differentiated SH-SY5Y cells and the NR uptake assay were selected to perform the subsequent experiments. For this purpose, the EC values were computed through the analysis of fitted NR concentration/response curves after 24 h of exposure to the drugs. The EC_20_, EC_50_, and EC_80_ values of each drug tested represent the concentrations that induce 20, 50, and 80% of cytotoxicity, respectively (Table 2).

### 3.2. Lipophilicity of 2C-T-X and 25TX-NBOMe Drugs and Its Correlation with Their Cytotoxicity

The lipophilicity of 2C-T-X/25TX-NBOMe drugs was evaluated by determining CHI values at pH = 2.3, obtained by comparing the retention times of the tested drugs with those of the reference compounds with reported CHI values (Supplementary Table S2). After applying Equation 1, the CHILogD_2.3_ showed a correlation between the lipophilicity, type of moieties presented in the thioether group, and the presence or absence of 2-methoxybenzyl group onto the nitrogen of the phenethylamine backbone. Generally, the insertion of 2-methoxybenzyl group increases the lipophilicity of the respective 2C-T-X drug. Furthermore, the lipophilicity of both families of drugs increases by increasing the number of carbons (thioethyl < thiopropyl group) and the conformation (thioisopropyl < thiopropyl group) of the alkyl chain.

**Figure 5:**
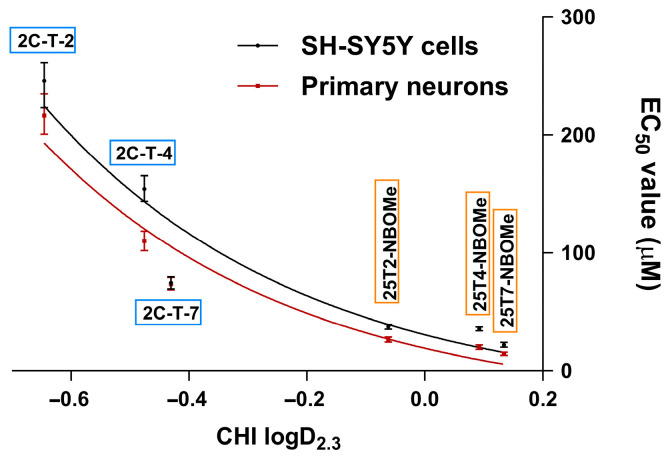
Correlations between the half maximal effective concentration (EC_50_) values with 95% confidence interval (CI) of 2C-T-X and 25TX-NBOMe drugs, obtained from lysosomal activity measurements (NR uptake assay) in both cell models, with the lipophilicity data.

By analyzing the data derived from measurements of lysosomal activity in both cell lines (NR uptake assay) and assessing the lipophilicity of the drugs under investigation, we were able to establish structure/property/cytotoxicity relationships. As observed in Figure 5, a clear correlation was observed between the lipophilicity of the drugs and the EC_50_ values obtained in cell viability assays for both cell lines. The data demonstrated that the drugs with higher lipophilicity exhibited lower EC_50_ values, revealing an inverse logarithmic correlation between these two parameters.

### 3.3. 2C-T-X and 25TX-NBOMe Drugs Significantly Induce Mitochondrial Membrane Depolarization in Differentiated SH-SY5Y Cells

**Figure 6:**
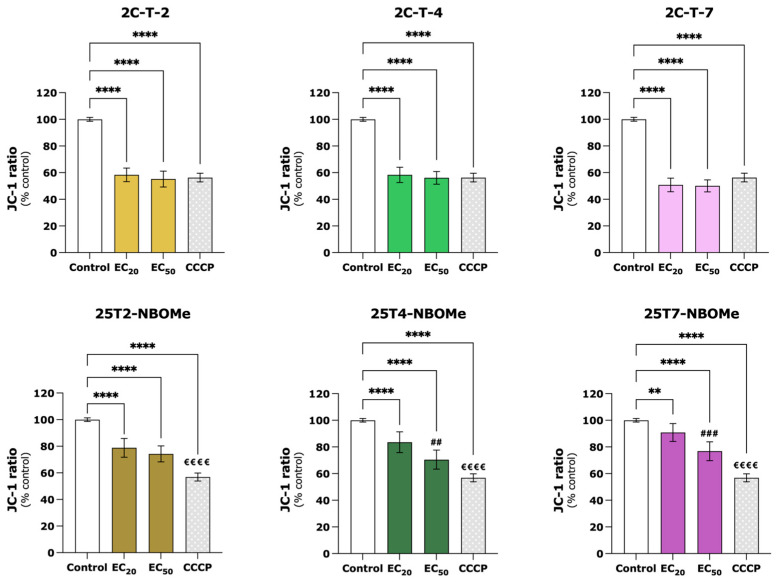
Mitochondrial membrane potential, estimated with the fluorescent JC-1 probe, in the differentiated SH-SY5Y cells, following 24 h of incubation with the EC_20_ and EC_50_ of the 2C-T-X and 25TX-NBOMe drugs and after 2 h of exposure to the positive control—CCCP (carbonyl cyanide m-chlorophenyl hydrazone, 100 µM). The results were computed as red/green fluorescence intensity ratio, expressed as a percentage of the control cells, and depicted as mean with a 95% confidence interval (CI) from a minimum of 5 independent experiments (3 replicates each). Statistical analysis was conducted using one-way ANOVA followed by Tukey’s multiple comparisons test. In all cases, *p* values below 0.05 were considered significant [** *p* < 0.01, **** *p* < 0.0001 drugs vs. control (0 μM); ## *p* < 0.01, ### *p* < 0.001 EC_20_ vs. EC_50_; €€€€ *p* < 0.0001 EC_20_ or EC_50_ vs. CCCP].

The mitochondrial membrane potential (ΔΨm) was evaluated using the JC-1 dye after the exposure of the differentiated SH-SY5Y cells to the EC_20_ and EC_50_ of the drugs under study, for 24 h. As depicted in Figure 6, all the drugs significantly decreased the red/green fluorescence ratio, indicating mitochondrial membrane depolarization. This effect was more pronounced for the 2C-T-X drugs; however, no significant differences were observed between EC_20_ and EC_50_. When compared to the control cells (100.0%), the EC_20_ and EC_50_ of 2C-T-2, 2C-T-4, and 2C-T-7 induced a significant decrease in mitochondrial membrane polarization to 58.4% and 55.2%, 58.3% and 56.1%, and 50.8% and 50.0% of the control cells, respectively. A similar depolarization was observed for the positive control CCCP (56.3%). Additionally, for the 25TX-NBOMe drugs, a concentration-dependent effect was observed. When compared to the control cells (100.0%), the EC_20_ and EC_50_ of 25T2-NBOMe, 25T4-NBOMe, and 25T7-NBOMe induced a significant decrease in mitochondrial membrane polarization to 78.7% and 74.3%, 83.5% and 70.5%, and 90.8% and 76.8% of the control cells, respectively.

### 3.4. 2C-T-X and 25TX-NBOMe Drugs Significantly Reduce Intracellular ATP Levels in Differentiated SH-SY5Y Cells

**Figure 7:**
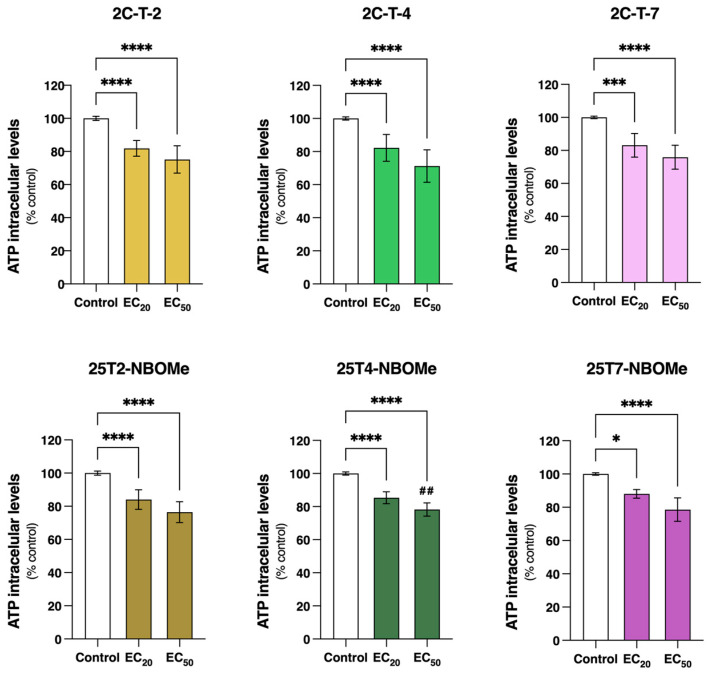
Intracellular levels of adenosine triphosphate (ATP), estimated through an ATP bioluminescence assay (luciferin-luciferase), in the differentiated SH-SY5Y cells, following 24 h of incubation with the EC_20_ and EC_50_ of the 2C-T-X and 25TX-NBOMe drugs. The results are depicted as mean with a 95% confidence interval (CI) from a minimum of 6 independent experiments (2 replicates each). Statistical analysis was conducted using one-way ANOVA followed by Tukey’s multiple comparisons test. In all cases, *p* values below 0.05 were considered significant [* *p* < 0.05, *** *p* < 0.001, **** *p* < 0.0001 vs. control (0 μM); ## *p* < 0.01 EC_20_ vs. EC_50_].

The intracellular ATP levels were evaluated by a luciferin/luciferase bioluminescence assay following the differentiated SH-SY5Y cells’ exposure to the EC20 and EC50 of the drugs for 24 h. As observed in Figure 7, all the drugs significantly reduced intracellular ATP levels. Similar results were observed between the 2C-T-X and the 25TX-NBOMe drugs, with the drugs’ EC_20_ significantly decreasing the intracellular ATP levels by approximately 10–20% of the control cells (2C-T-2: 18.1%; 25T2-NBOMe: 16.0%; 2C-T-4: 17.8%; 25T4-NBOMe: 14.6%; 2C-T-7: 16.9% and 25T7-NBOMe: 12.0%). With the exception of 25T4-NBOMe, no significant differences in the ATP levels were observed between the EC_20_ and EC_50_ of the drugs. However, the EC_50_ of the drugs significantly decreased the intracellular ATP levels by approximately 20–30% when compared to the control cells (2C-T-2: 24.8%; 25T2-NBOMe: 23.5%; 2C-T-4: 28.7%; 25T4-NBOMe: 21.8%; 2C-T-7: 24.1% and 25T7-NBOMe: 21.4%).

### 3.5. 2C-T-7 and 25T7-NBOMe Significantly and “Immediately” Increase Intracellular Calcium Levels in a Concentration-Dependent Manner in Differentiated SH-SY5Y Cells

**Figure 8:**
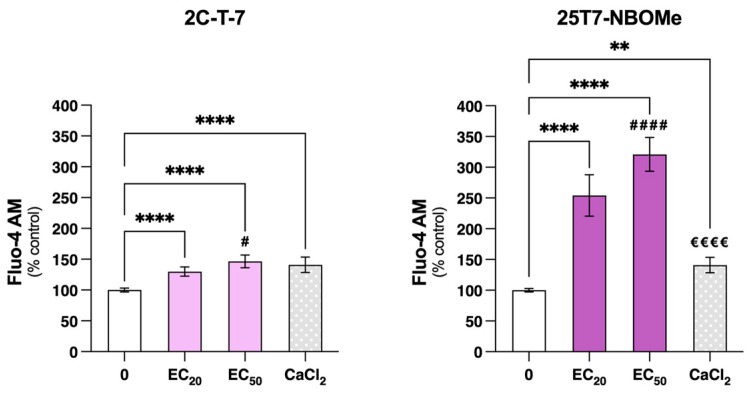
Ca^2^⁺ intracellular levels, evaluated with the Fluo-4 AM probe, in the differentiated SH-SY5Y cells after immediate exposure to the EC_20_ and EC_50_ of 2C-T-7 and 25T7-NBOMe and to the positive control—CaCl_2_ (25 mM). The results are depicted as mean with a 95% confidence interval (CI) from a minimum of 4 independent experiments (3 replicates each). Statistical analysis was conducted using one-way ANOVA followed by Tukey’s multiple comparisons test. In all cases, *p* values below 0.05 were considered significant [** *p* < 0.01, **** *p* < 0.0001 vs. control (0 μM); # *p* < 0.05, #### *p* < 0.0001 EC_20_ vs. EC_50_; €€€€ *p* < 0.0001 EC_20_ or EC_50_ vs. CaCl_2_].

Since the experiments of ΔΨm and intracellular ATP levels evidenced similar results for all pairs of drugs, the most cytotoxic pair was chosen to evaluate their effect on intracellular Ca^2^⁺ levels. Thus, the Ca^2^⁺ levels were monitored in real-time by fluorescence microscopy using the Fluo-4 AM probe immediately after the exposure of the differentiated SH-SY5Y cells to the EC_20_ and EC_50_ of 2C-T-7 and 25T7-NBOMe. As depicted in Figure 8, both drugs significantly increased the intracellular Ca^2^⁺ levels in a concentration-dependent manner, demonstrating a relevant intracellular Ca^2^⁺ mobilization. In comparison to the control cells (100.0%), both the EC_20_ and the EC_50_ of 2C-T-7 resulted in a significant and immediate increase in Ca^2^⁺ intracellular levels to 129.9% and 146.5%, respectively, which was similar to those observed for the positive control CaCl_2_ (141.0%). Regarding 25T7-NBOMe, the increase in Ca^2^⁺ intracellular levels was even more remarkable than that observed for 2C-T-7. When compared to the control cells (100.0%), the EC_20_ and the EC_50_ of 25T7-NBOMe significantly increased the Ca^2^⁺ intracellular levels to 254.2% and 320.9%, respectively. Moreover, a significant difference between the EC_20_ and EC_50_ of 25T7-NBOMe was observed.

### 3.6. 2C-T-X and 25TX-NBOMe Drugs Do Not Change Reactive Species Intracellular Levels in SH-SY5Y Cells

**Figure 9:**
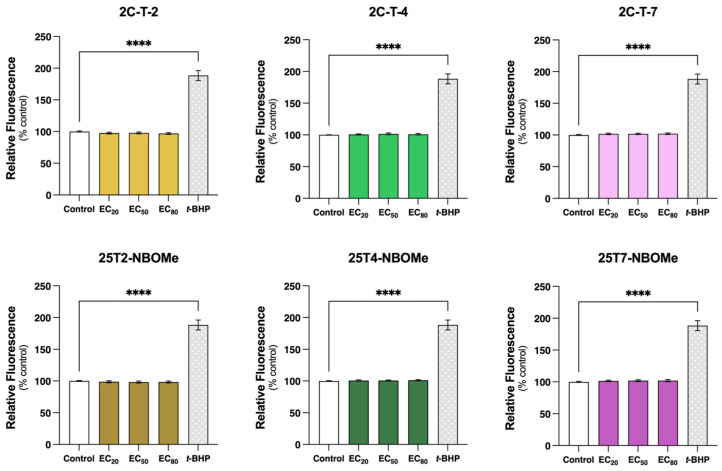
Intracellular levels of reactive oxygen species (ROS) and reactive nitrogen species (RNS), estimated with the DCFH-DA probe (10 μM), in the differentiated SH-SY5Y cells, following 24 h of incubation with the EC_20_, EC_50_, and EC_80_ of the 2C-T-X and 25TX-NBOMe drugs and to the positive control tert-butyl hydroperoxide (t-BHP, 200 µM, 24 h). The results are depicted as mean with a 95% confidence interval (CI) from a minimum of 5 independent experiments (3 replicates each). Statistical comparisons were obtained using one-way ANOVA followed by Dunnett’s multiple comparisons test. In all cases, *p* values below 0.05 were considered significant [**** *p* < 0.0001 vs. control (0 μM)].

The intracellular ROS/RNS levels were evaluated using the DCFH-DA probe following the exposure of the differentiated SH-SY5Y cells to the EC_20_, EC_50,_ and EC_80_ of the drugs for 24 h. As observed in Figure 9, none of the drugs, at any of the tested concentrations, significantly altered the intracellular ROS/RNS levels. However, the positive control—*t*-BHP (100 µM, 24 h)—significantly increased ROS levels by approximately 90% (*t*-BHP-treated cells 188.4% vs. control cells 100.0%).

### 3.7. 2C-T-X and 25TX-NBOMe Drugs Significantly Deplete Intracellular Total Glutathione (tGSH) Levels in Differentiated SH-SY5Y Cells

**Figure 10:**
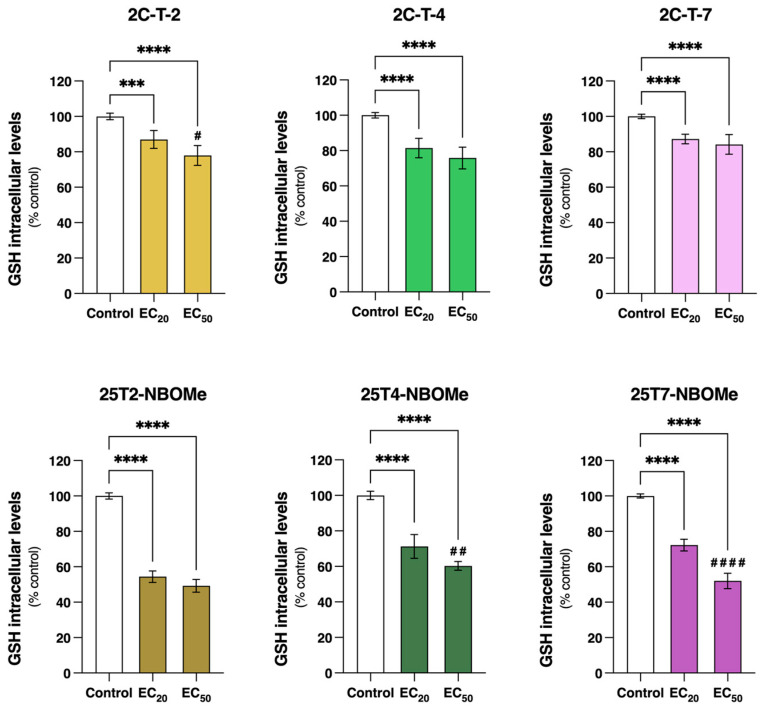
Intracellular total glutathione (tGSH) levels, estimated through the DTNB-GSH recycling assay, in the differentiated SH-SY5Y cells, following 24 h of incubation with the EC_20_ and EC_50_ of the 2C-T-X and 25TX-NBOMe drugs. The results are depicted as mean with a 95% confidence interval (CI) from a minimum of 7 independent experiments (2 replicates each). Statistical analysis was conducted using one-way ANOVA followed by Tukey’s multiple comparisons test. In all cases, *p* values below 0.05 were considered significant [*** *p* < 0.001, **** *p* < 0.0001 vs. control (0 μM); # *p* < 0.05, ## *p* < 0.01, #### *p* < 0.0001 EC_20_ vs. EC_50_].

The extracellular and intracellular tGSH levels were quantified through the DTNB-GSSG reductase-recycling assay following the exposure of the differentiated SH-SY5Y cells to the EC_20_ and EC_50_ of the drugs for 24 h. In these experimental conditions, the extracellular tGSH levels were below the detection limit of the method for all the tested drugs. Thus, only the intracellular tGSH levels are depicted in Figure 10. All the drugs significantly reduced the intracellular tGSH levels of the SH-SY5Y cells in a concentration-dependent manner. When compared to the control cells (100.0%), the EC_20_ and EC_50_ of 2C-T-2, 2C-T-4, and 2C-T-7 significantly decreased the intracellular tGSH levels to 87.0% and 80.0%, 81.4% and 75.8%, and 84.2% and 82.4%, respectively. This effect was more pronounced for the 25TX-NBOMe drugs. The EC_20_ and EC_50_ of 25T2-NBOMe, 25T4-NBOMe, and 25T7-NBOMe significantly decreased intracellular tGSH levels to 54.4% and 49.2%, 71.2% and 60.3%, and 72.2% and 52.0% of the control cells, respectively. Furthermore, for 2C-T-2, 25T4-NBOMe, and 25T7-NBOMe, a significant difference between the EC_20_ and EC_50_ was observed.

### 3.8. Inhibition of Glutamate-Cysteine Ligase Does Not Change the Drugs-Induced Cytotoxicity in Differentiated SH-SY5Y Cells

To further elucidate the role of GSH on drugs’ cytotoxicity, the impact of GCL (enzyme required in the first step of glutathione synthesis) inhibition with BSO was explored. Initially, BSO (100 μM) cytotoxicity and its ability to deplete intracellular tGSH levels were evaluated in the differentiated SH-SY5Y cells after 24 h of exposure. No significant cytotoxic effects were observed (Supplementary Figure S2); however, a significant depletion of GSH intracellular levels was detected (Supplementary Figure S3, control cells: 100.0% vs. cells exposed to BSO: 9.2%). Then, the impact of GSH intracellular levels on the drugs’ cytotoxicity was evaluated through the GCL inhibition after 24 h of simultaneous exposure to BSO and the drugs EC_50_ and EC_80_. Despite the impact of BSO on glutathione intracellular levels (Supplementary Figure S3), as observed in Supplementary Figure S4, no significant differences were observed between the untreated cells and the cells pretreated with BSO in what concerns drug-induced cytotoxicity.

### 3.9. Metabolism via Monoamine Oxidases (MAO) Does Not Seem to Play a Significant Role in Drugs-Induced Cytotoxicity in Differentiated SH-SY5Y Cells

To better understand the role of MAO metabolism in the cytotoxicity of these drugs, the impact of MAO-A and MAO-B inhibition, with clorgyline and rasagiline, respectively, was investigated. Neither clorgyline nor rasagiline (1 μM) showed significant cytotoxicity towards the SH-SY5Y cells after 24 h of exposure (Supplementary Figure S5). Thus, this concentration was selected for co-incubation experiments, where the impact of MAO inhibition on drugs’ cytotoxicity was evaluated after 24 h of simultaneous exposure to clorgyline or rasagiline (1 μM) and the EC_50_ or EC_80_ of the drugs. As portrayed in Supplementary Figure S6, MAO-A or MAO-B co-inhibition had no significant effect on the cytotoxicity of the drugs.

### 3.10. 25TX-NBOMe Drugs Seem to Have Inhibitory Activity on MAO-B

**Figure 11:**
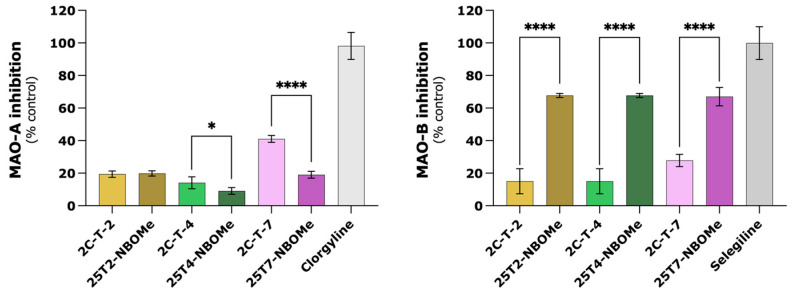
Evaluation of human monoamine oxidase (hMAO-A and hMAO-B) inhibitory activities of 2C-T-X (10 μM) and 25TX-NBOMe (10 μM) drugs and reference inhibitors: clorgyline (MAO-A inhibitor) and selegiline (MAO-B inhibitor). The results are depicted as mean with a 95% confidence interval (CI) of 3 independent experiments (3 replicates each). Statistical analysis was conducted using two-way ANOVA followed by Tukey’s multiple comparisons test. In all cases, *p* values below 0.05 were considered significant [* *p* < 0.05, **** *p* < 0.0001, 2C-T-X vs. 25TX-NBOMe].

The inhibitory activity of 2C-T-X and 25TX-NBOMe drugs towards MAO isoforms was then evaluated in a cell-free assay. Accordingly, drugs at 10 µM were incubated with human recombinant MAO-A or MAO-B (hMAO-A and hMAO-B, respectively) and with kynuramine, a non-selective MAO substrate. The data obtained are depicted in Figure 11. The percentages of the inhibition of clorgyline and selegiline at 10 µM towards hMAO-A and hMAO-B were 98.2% and 99.9%, respectively. The results clearly showed that 2C-T-X and 25TX-NBOMe drugs are not potent hMAO inhibitors. Unlike the reference inhibitors, the drugs under study did not completely inhibit the activity of hMAO isoforms at a concentration of 10 µM. Concerning hMAO-A, all the drugs showed percentages of inhibition between 9.1% and 41%, with 2C-T-X drugs displaying similar-to-higher hMAO-A inhibitory activity than the 25TX-NBOMe counterparts. A different trend was observed for the MAO-B isoform: the 25TX-NBOMe drugs were stronger hMAO-B inhibitors than the related 2C-T-X analogs, presenting percentages of inhibition close to 70%, while 2C-T-X drugs only showed percentages of inhibition between 15.0% and 27.8%.

## 4. Discussion

For some years, NPS, including many phenethylamine derivatives, were legal alternatives to illicit drugs. Although over time these drugs lost their legal status and are currently strictly regulated [37,38,39], they are still available on online platforms and on the streets, posing significant risks associated with their use [40,41]. Since the brain is a target organ of these drugs, understanding their neurotoxic profile is of utmost importance, specially to minimize their potential harm. Previous studies, in vivo and in vitro, demonstrated the neurotoxicity of synthetic phenethylamines, with acute intoxications and fatalities being reported [10,11,12,21,42,43]. Nonetheless, to the best of our knowledge, there is only one published study regarding specifically the neurotoxicity of 2C-T-2, 2C-T-4, and 2C-T-7 [42], and no studies regarding 25T2-NBOMe, 25T4-NBOMe, and 25T7-NBOMe. Indeed, as far as we know, this is the first study to investigate the cytotoxicity of these NBOMe drugs.

In this study, we initially evaluated the ability of the tested drugs to induce cytotoxicity in differentiated SH-SY5Y cells and in primary rat cortical cultures. Twenty-four hours of exposure to the drugs resulted in a concentration-dependent lysosomal impairment, as evaluated by the NR uptake assay (for both models, Figure 3 and Figure 4), and a significant mitochondrial dysfunction, as evaluated by the MTT reduction assay (evaluated only in the SH-SY5Y cells, Figure 3). All the 25TX-NBOMe drugs were more cytotoxic than their corresponding 2C-T-X drugs (Table 1). Consistent with their lipophilicity (higher lipophilicity is directly correlated with higher cytotoxicity), the drugs can be ranked from least to most cytotoxic as follows: 2C-T-2 < 2C-T-4 < 2C-T-7 < 25T2-NBOMe < 25T4-NBOMe < 25T7-NBOMe (Figure 5), which agrees with the severity of the case reports for NBOMe drug intoxications [14,43,44]. Structure/cytotoxicity relationships revealed that increasing the number of carbons in the alkyl chain (thioethyl < thiopropyl) and changing their conformation from thioisopropyl to thiopropyl significantly increased the lipophilicity and cytotoxicity of the drugs. Using different in vitro models—dopaminergic CATH.a cells and 5-HT-containing B65 cells—Asanuma et al. also investigated the neurotoxic profile of 2C-T-2, 2C-T-4, and 2C-T-7. They showed that, following a 24 h exposure, all drugs (0–250 μM) induced a concentration-dependent cytotoxic effect, as evaluated by the lactate dehydrogenase (LDH) assay. In agreement with our results, 2C-T-7 was identified as the most cytotoxic drug. However, contrary to what we observed, 2C-T-2 was more cytotoxic than 2C-T-4. Moreover, the group showed that 2C-T-X drugs were significantly more cytotoxic than MDMA (3,4-methylenedioxymethamphetamine) and methamphetamine (EC_50_ ranging from 1.5–3 mM for MDMA and methamphetamine vs. EC_50_ ranging from 100 to 300 µM for 2C-T-X drugs), demonstrating their high toxic potential [42].

Given the high energy demand of the brain, whose ATP is mostly produced by mitochondrial respiration, it becomes particularly susceptible to mitochondrial dysfunction [45,46]. The gradient established by the active pumping of protons through the electron transport chain results in the storage of potential energy that generates the ΔΨm. ATP synthase, an enzyme present in the mitochondrial inner membrane, uses the flow of protons back into the matrix to drive the synthesis of ATP from adenosine diphosphate (ADP) and inorganic phosphate (Pi) [47,48]. Thus, maintaining a stable ΔΨm is crucial not only for efficient energy production but also for maintaining cellular health [49]. We observed that after 24 h of exposure, all the tested drugs (EC_20_ and EC_50_) induced a significant mitochondrial membrane depolarization s and decreased the intracellular ATP levels (Figure 6 and Figure 7), evidencing mitochondrial impairment. Similar results have been reported for other drugs [50,51], including drugs from the 2C and NBOMe families [21].

Ca^2+^ homeostasis is vital for normal cell functioning, as it serves as a secondary messenger in many signaling pathways, including neurotransmitter release, muscle contraction, and programmed cell death [52,53]. To uphold its homeostasis, Ca^2^⁺ movement in and out of cells and organelles is meticulously regulated by a network of transporters and buffering systems [54]. Although the major intracellular Ca^2^⁺ storage organelle is the endoplasmic reticulum (ER), mitochondria can also store this ion [55]. Importantly, high cellular Ca^2^⁺ concentrations during a long period of time can result in cell death [56]. In our study, we observed, for 2C-T-7 and 25T7-NBOMe, a concentration-dependent increase in intracellular Ca^2^⁺ levels (Figure 8). This is indicative of an increased intracellular Ca^2+^ mobilization and the consequent deregulation of Ca^2^⁺ homeostasis. This effect was more pronounced for the NBOMe drug, with the EC_20_ and the EC_50_ of 25T7-NBOMe increasing Ca^2^⁺ levels to over 250 and 300% of the control cells, respectively. Noteworthy, this effect was observed immediately after cell exposure to the tested drugs, highlighting the impacts of these drugs on Ca^2^⁺ levels and how fast they occur. Thus, it is possible that changes in the intracellular Ca^2^⁺ levels precede the observed mitochondrial membrane depolarization and decline in ATP levels, emphasizing mitochondrial dysfunction as an important pathway for these drugs-induced cytotoxic effects.

There is a complex interplay involving calcium, ATP, and ROS, mostly centered around the mitochondria [57]. ROS are naturally produced in cells, especially during mitochondrial respiration. In small amounts, ROS are important for cell signaling and regulation; however, when ROS production is excessive and/or if cells are not able to efficiently inactivate them, they can lead to oxidative stress [58,59]. Although oxidative stress has been reported as an impacting factor in the toxicity of different drugs [50,51,60], we observed that none of our tested drugs changed intracellular reactive species formation in the differentiated SH-SY5Y cells, even at high concentrations (EC_80_) (Figure 9). Thus, in our model, oxidative stress seems to have little to no relevance in the cytotoxicity of these drugs. Nevertheless, using a different model (serotonergic B65 cells) and method (MitoTracker red CM-H2XRos), Asanuma et al. reported an increase in the mitochondrial production of ROS following a 3 h exposure to 50 μM 2C-I, 2C-T-2, and 2C-T-7, but no significant alterations were detected for 2C-C and 2C-T-4 [42]. Additionally, Cocchi et al. identified ROS production as a genotoxicity mechanism in TK6 cells treated for 1 h with 35 µM 2C-H, 35 µM 2C-I, 12.5 µM 2C-B, and 12.5 µM 25B-NBOMe (about a two-fold increase in ROS levels was observed for all drugs) [61]. As perceived, some discrepancy regarding the role of oxidative stress in the cytotoxicity mechanism of these drugs still endures. Therefore, more studies are needed to fully elucidate how oxidative stress contributes to the toxic effect of these drugs. GSH is a powerful antioxidant, essential for ROS scavenging, detoxification, and the elimination of xenobiotics [62]. Although no significant alterations in reactive species formation were observed (Figure 9), our data demonstrated a significant reduction in intracellular tGSH content following a 24 h incubation with all tested drugs (Figure 10). Thus, this decrease in the tGSH content suggests that the drugs might be forming direct conjugates with glutathione. Interestingly, the inhibition of GCL, the enzyme required for the first step of glutathione synthesis, did not impact the drugs’ cytotoxicity (Figure S4), suggesting that glutathione does not play a central role in the detoxification of these drugs. Similar results were described for other NPS, including piperazines (BZP, TFMPP, MeOPP, and MDBP) and phenethylamines (mescaline, 2C-B, 2C-N, mescaline-NBOMe, 25B-NBOMe, and 25C-NBOMe). In agreement with our results, and using the same in vitro model, both studies showed a significant decrease in the tGSH content with no ROS production [21,63].

Furthermore, the impact of MAO-A and MAO-B inhibition, by clorgyline and rasagiline, respectively, in drug-induced cytotoxicity was also explored in the differentiated SH-SY5Y cells. However, the metabolism via MAO does not seem to play a significant role in the cytotoxicity of the tested drugs (Figure S6). Using the same experimental conditions, we recently reported that MAO-mediated metabolism mainly functions as a potential detoxification pathway for mescaline, 2C-B, 2C-N, mescaline-NBOMe, and 25N-NBOMe, since a significant increase in the drug-induced cytotoxicity was observed upon MAO inhibition [21]. Previously, Theobald et al. studied the involvement of MAO-A and MAO-B in the deamination (crucial metabolic step for 2C-X drugs) of a group of 2C drugs (2C-B, 2C-I, 2C-D, 2C-E, 2C-T-2, and 2C-T-7) in a rat model. They showed that both isoenzymes can catalyze the deamination of these drugs. Therefore, 2C-X drugs might be susceptible to drug-drug interactions with MAO inhibitors, feasibly leading to higher plasma concentrations of these drugs, raising the possibility of toxic side effects [64]. However, as formerly described, this was not observed in our in vitro model. A possible explanation for these results may be the fact that the resulting metabolites present similar cytotoxic effects to those of the parent drug (Theobald et al. showed that at least 2C-T-2 and 2C-T-7 are metabolized by MAO [64]). Additionally, we also assessed the impact of the tested drugs on MAO activity in a cell-free system using recombinant *h*MAO-A and *h*MAO-B, along with kynuramine, a non-selective MAO substrate. When compared to reference inhibitors (clorgyline and selegiline for MAO-A and MAO-B, respectively), the tested drugs were not fully inhibited (Figure 11). Of note, *h*MAO-B had the best inhibitory results, especially for the 25TX-NBOMe drugs. Overall, we concluded that MAO metabolism exhibits limited impact on the drugs-induced cytotoxicity and the studied drugs are poor *h*MAO inhibitors.

## 5. Conclusions

The consumption of phenethylamine derivatives still presents notable public health concerns, especially due to a lack of scientific knowledge regarding these substances. In this study, we explored the pathways involved in the neurotoxic effects induced by 2C-T-2, 2C-T-4, and 2C-T-7, and their corresponding NBOMes. In line with previously reported case studies and our own findings on lipophilicity, this in vitro study demonstrated that NBOMe drugs are remarkably more cytotoxic than their 2C-T-X counterparts. Furthermore, the cytotoxicity of these drugs was directly associated with mitochondrial dysfunction, as evidenced by the loss of mitochondrial membrane potential and reduction in intracellular ATP levels. Intracellular Ca^2^⁺ deregulation was also observed for 2C-T-7 and 25T7-NBOMe, suggesting a disruption of Ca^2^⁺ homeostasis. Interestingly, while no significant alteration in reactive species formation was observed, a decrease in the intracellular tGSH content was noted. Overall, these results increase our understanding regarding these drugs, with an emphasis on the mechanisms underlying their neurotoxicity.

## Figures and Tables

**Table 1:** Half maximal effective concentration (EC_50_) values of 2C-T-X and 25TX-NBOMe drugs, obtained from concentration/response curves in differentiated SH-SY5Y cells and in primary rat cortical neurons after 24 h of exposure.

	SH-SY5Y Cells		Primary Rat Cortical Cultures
	NR EC_50_ Values (μM)	MTT EC_50_ Values (μM)	NR EC_50_ Values (μM)
2C-T-2	245.9 (223.2 to 261.3)	305.5 (294.5 to 317.3)	216.4 (200.6 to 234.9)
25T2-NBOMe	37.4 **** (35.4 to 39.5)	46.8 **** (45.2 to 48.4)	26.5 **** (24.4 to 28.7)
2C-T-4	154.1 (143.7 to 165.5)	190.7 (182.9 to 199.5)	110.2 (102.1 to 118.5)
25T4-NBOMe	35.7 **** (34.0 to 37.2)	36.0 **** (34.5 to 37.6)	19.9 **** (18.2 to 21.8)
2C-T-7	74.5 (69.4 to 79.8)	92.3 (75.3 to 100.8)	73.6 (68.6 to 79.1)
25T7-NBOMe	21.8 **** (21.4 to 22.3)	23.3 **** (22.7 to 23.9)	14.4 **** (13.1 to 15.8)

The concentration/response curves were fitted using the least squares method, and the comparisons between the 2C-T-X and the 25TX-NBOMe curves were reached employing the extra sum-of-squares F test. The results are presented as mean with a 95% confidence interval (CI). In all cases, *p* values below 0.05 were considered significant (**** *p* < 0.0001 2C-T-X vs. 25TX-NBOMe).

**Table 2:** Concentrations of the 2C-T-X and 25TX-NBOMe drugs inducing 20, 50, and 80% of cytotoxicity (EC_20_, EC_50_, and EC_80_, respectively) in the differentiated SH-SY5Y cells, assessed by the NR uptake assay, following 24 h of incubation.

	SH-SY5Y Cells NR EC Values (μM)		
	EC_20_	EC_50_	EC_80_
2C-T-2	123.2 (107.1 to 138.8)	245.9 (223.2 to 261.3)	491.0 (413.7 to 514.4)
25T2-NBOMe	22.2 **** (20.3 to 24.4)	37.4 **** (35.4 to 39.5)	62.9 **** (58.8 to 67.7)
2C-T-4	81.5 (79.9 to 92.1)	154.1 (143.7 to 165.5)	291.4 (255.9 to 342.3)
25T4-NBOMe	22.1 **** (20.1 to 23.8)	35.7 **** (34.0 to 37.2)	57.8 **** (54.4 to 59.6)
2C-T-7	39.5 (34.4 to 44.4)	74.5 (69.4 to 79.8)	140.5 (125.4 to 161.2)
25T7-NBOMe	16.4 **** (15.6 to 17.2)	21.8 **** (21.4 to 22.3)	28.9 **** (27.5 to 30.5)

Neutral red (NR) concentration/response curves were fitted using the least squares method, and the comparisons between the 2C-T-X and the 25TX-NBOMe curves were estimated with the extra sum-of-squares F test. The results are presented as mean with a 95% confidence interval (CI). In all cases, *p* values below 0.05 were considered significant (**** *p* < 0.0001 2C-T-X vs. 25TX-NBOMe).

## Data Availability

The data that support the findings of this study are available from the corresponding author upon reasonable request.

## Supplementary Materials

The following supporting information can be downloaded at: https://www.mdpi.com/article/10.3390/jox14020044/s1. Table S1: EC_50_ (half-maximum-effect concentrations), Top (maximal effect), Bottom (baseline) and Hill Slope values of the 2C-T-X and 25TX-NBOMe concentration-response curves in differentiated SH-SY5Y cells (NR uptake and MTT reduction assays) and in primary rat cortical cultures (NR uptake assay), after 24 h of exposure. Table S2: Calibration set of compounds for the chromatographic hydrophobicity index (CHI) lipophilicity determination at pH 2.3. Figure S1: Calibration curve obtained after plotting the retention times of the test mixture of compounds against the CHI values at pH 2.3 [16]. Figure S2: L-buthionine sulfoximine (BSO, 100 μM for 24 h + 50 μM for an additional 24 h) cytotoxicity in differentiated SH-SY5Y cells evaluated by the NR uptake assay. Results are presented as mean with 95 % confidence interval (CI) from 3 independent experiments (at least 4 replicates each). Statistical comparisons were obtained using one-way ANOVA, followed by the Dunnett’s multiple comparisons test. Figure S3: Intracellular glutathione (GSH) levels, evaluated through DTNB-GSH recycling assay, in differentiated SH-SY5Y cells, after 24 h of exposure to L-buthionine sulfoximine (BSO, 100 μM). Results are presented as mean with 95% confidence interval (CI) from 3 independent experiments (at least 4 replicates each). Statistical comparisons were made using the unpaired *t* test [**** *p* < 0.0001 vs control (0 μM)]. Figure S4: Impact of glutamate-cysteine ligase (GCL, enzyme required in the first step of glutathione synthesis) inhibition on the cytotoxicity of 2C-T-X and 25TX-NBOMe (EC_50_ and EC_80_) drugs in differentiated SH-SY5Y cells. Cells were pre-exposed to L-buthionine sulfoximine (BSO, 100 μM) for 24 h, and then co-incubated with the drugs for an additional 24 h (50 μM BSO + drugs, in grey). Following the incubation period, the cytotoxicity was evaluated by the NR uptake assay. Results are presented as mean with 95% confidence interval (CI) from a minimum of 4 independent experiments (3 replicates each). Statistical analysis were obtained using two-way ANOVA followed by Šídák’s multiple comparisons test. Figure S5: Clorgyline and rasagiline (1 μM) cytotoxicity in differentiated SH-SY5Y cells evaluated by the NR uptake assay, after 24 h of incubation. Results are presented as mean with 95% confidence interval (CI) from at least 4 independent experiments (3 replicates each). Statistical comparisons were obtained using one-way ANOVA, followed by the Dunnett’s multiple comparisons test. Figure S6: Effect of monoamine oxidase (MAO) inhibition on the cytotoxicity of 2C-T-X (and 25TX-NBOMe (EC_50_ and EC_80_) drugs in differentiated SH-SY5Y cells, following 24 h of incubation with EC_20_ and EC_50_ of 2C-T-X and 25TX-NBOMe drugs, in the presence or absence of two pre-exposed (for 1 h) MAO inhibitors: 1 μM clorgyline (MAO-A inhibitor, light gray) or 1 μM rasagiline (MAO-B inhibitor, dark grey). Following the incubation period, the cytotoxicity was evaluated by the NR uptake assay. Results are presented as mean with 95% confidence interval (CI) from a minimum of 4 independent experiments (3 replicates each). Statistical analisys were obtained using two-way ANOVA followed by Tukey’s multiple comparisons test.
