# Supplementary material for: Mechanistic Insights into the Neurotoxicity of 2,5-Dimethoxyphenethylamines (2C) and Corresponding N-(2-methoxybenzyl)phenethylamine (NBOMe) Drugs
Source: J Xenobiot. 2024 Jun 5;14(2):772–97. doi: 10.3390/jox14020044 (PMC11204507; doi:10.3390/jox14020044)
Supplement: Supplementary file 1 [file jox-14-00044-s001.zip › jox-2990329-supplementary.pdf]

Journal of Xenobiotics

MDPI

Supplementary Materials: Mechanistic Insights into the Neurotoxicity of 2,5-dimethoxyphenethylamines (2C) and Corresponding *N*-(2-methoxybenzyl)phenethylamine (NBOMe) Drugs

Eva Gil-Martins, Fernando Cagide-Fagín, Daniel Martins, Ana Borer, Daniel José Barbosa, Carlos Fernandes, Daniel Chavarria, Fernando Remião, Fernanda Borges and Renata Silva

Supplementary Table S1. EC_{50} (half-maximum-effect concentrations), Top (maximal effect), Bottom (baseline) and Hill Slope values of the 2C-T-X and 25TX-NBOMe concentration-response curves in differentiated SH-SY5Y cells (NR uptake and MTT reduction assays) and in primary rat cortical cultures (NR uptake assay), after 24 hours of exposure.

	Neutral Red SH-SY5Y cells					
	**2C-T-2**	**25T2-NBOMe**	**2C-T-4**	**25T4-NBOMe**	**2C-T-7**	**25T7-NBOMe**
EC_{50} [half-maximum-effect concentrations, μM (95% CI)]	245.9 (223.2 to 261.3)	37.4**** (35.4 to 39.5)	154.1 (143.7 to 165.5)	35.7**** (34.0 to 37.2)	74.5 (69.4 to 79.8)	21.8**** (21.4 to 22.3)
Top (maximal cell death, % control)	100.0	98.41	96.98	100.0	97.99	94.74
Bottom (baseline, % control)	4.054	1.962	5.069	4.881	6.754	8.660
Hill slope	2.005	2.667*	2.176	2.898****	2.184	4.908****
Curve *p* value (comparison between the fitted curves)	-	< 0.0001	-	< 0.0001	-	< 0.0001
	**MTT SH-SY5Y cells**					
	**2C-T-2**	**25T2-NBOMe**	**2C-T-4**	**25T4-NBOMe**	**2C-T-7**	**25T7-NBOMe**
EC_{50} [half-maximum-effect concentrations, μM (95% CI)]	305.5 (294.5 to 317.3)	46.8**** (45.2 to 48.4)	190.7 (182.9 to 199,5)	36.0**** (34.5 to 37.6)	92.3 (75.3 to 100.8)	23.3**** (22.7 to 23.9)
Top (maximal cell death, % control)	97.13	95.97	99.28	100.0	100.0	96.91
Bottom (baseline, % control)	4.516	3.123	4.106	1.753	5.625	2.444
Hill slope	5.038	8.094**	2.775	4.064****	1.819	6.980****
Curve *p* value (comparison between the fitted curves)	-	< 0.0001	-	< 0.0001	-	< 0.0001
	**Neutral Red Primary Rat Cortical Neurons**					
	**2C-T-2**	**25T2-NBOMe**	**2C-T-4**	**25T4-NBOMe**	**2C-T-7**	**25T7-NBOMe**
EC_{50} [half-maximum-effect concentrations, μM (95% CI)]	216.4 (200.6 to 234.9)	26.5**** (24.4 to 28.7)	110.2 (102.1 to 118.5)	19.9**** (18.2 to 21.8)	73.6 (68.6 to 79.1)	14.4**** (13.1 to 15.8)
Top (maximal cell death, % control)	83.85	81.45	82.56	83.51	84.63	82.95
Bottom (baseline, % control)	1.639	3.637	0.7855	2.059	1.452	3.972
Hill slope	3.138	3.102	2.808	2.526	2.560	2.557
Curve *p* value (comparison between the fitted curves)	-	< 0.0001	-	< 0.0001	-	< 0.0001

Concentration-response curves were fitted using least squares as the fitting method, the comparisons between the 2C-T-X and the 25TX-NBOMe curves were made using the extra sum-of-squares F test and the results are presented as mean with 95 % confidence interval (CI). In all cases, p values < 0.05 were considered significant (*$p < 0.05$, ** $p < 0.01$, **** $p < 0.0001$ for 2C-T-X *vs* 25TX-NBOMe).

Supplementary Table S2. Calibration set of compounds for the chromatographic hydrophobicity index (CHI) lipophilicity determination at pH 2.3.

Reference compounds	t_r (min)	CHI_0 pH 2.3
Benzimidazole	4.484	6.3
Theophyline	4.601	17.9
Paracetamol	4.804	18.77
Caffeine	5.631	23.41
Colchicine	6.363	43.9
Carbamazepine	6.969	60.42
Indole	7.310	72.1
Propiophenone	7.776	77.4
Butyrophenone	8.497	87.3

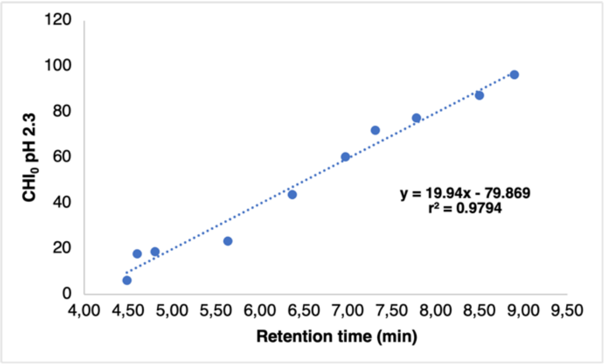

Supplementary Figure S1. Calibration curve obtained after plotting the retention times of the test mixture of compounds against the CHI values at pH 2.3 [16]

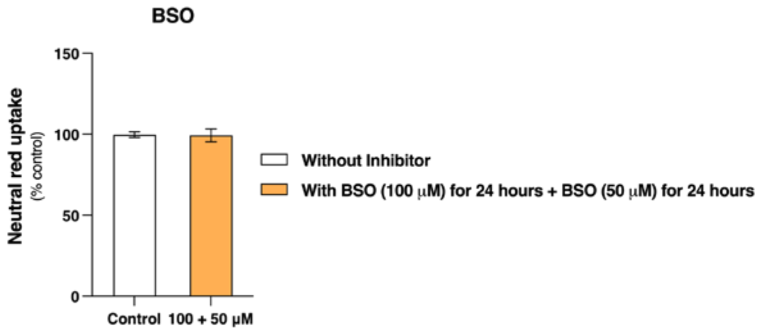

Supplementary Figure S2. L-buthionine sulfoximine (BSO, 100 μM for 24 hours + 50 μM for an additional 24 hours) cytotoxicity in differentiated SH-SY5Y cells evaluated by the NR uptake assay. Results are presented as mean with 95 % confidence interval (CI) from 3 independent experiments (at least 4 replicates each). Statistical comparisons were obtained using one-way ANOVA, followed by the Dunnett's multiple comparisons test.

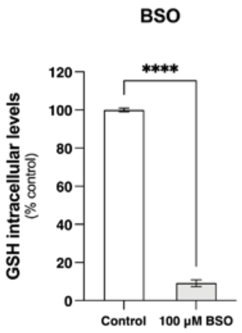

Supplementary Figure S3. Intracellular glutathione (GSH) levels, evaluated through DTNB-GSH recycling assay, in differentiated SH-SY5Y cells, after 24 hours of exposure to L-buthionine sulfoximine (BSO, 100 µM). Results are presented as mean with 95 % confidence interval (CI) from 3 independent experiments (at least 4 replicates each). Statistical comparisons were made using the unpaired t test [**** $p < 0.0001$ vs control (0 µM).

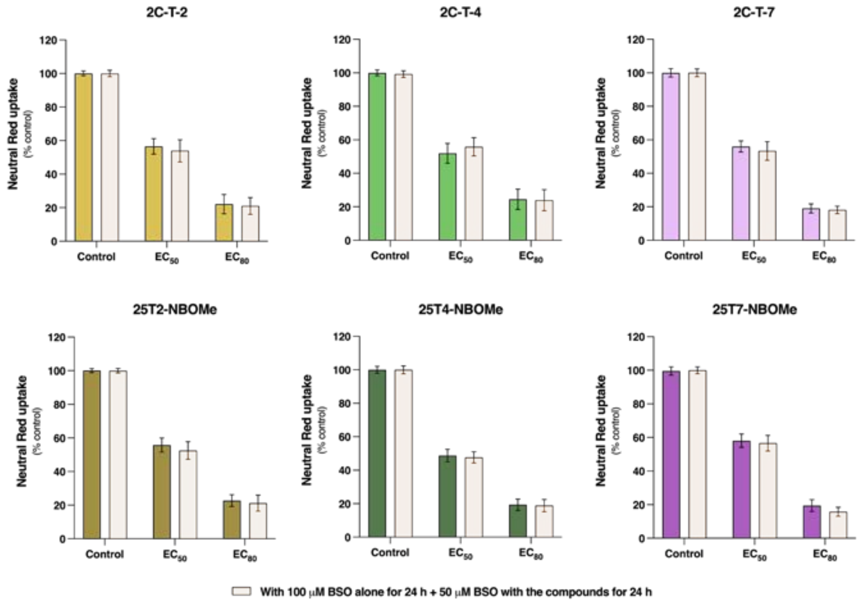

Supplementary Figure S4. Impact of glutamate-cysteine ligase (GCL, enzyme required in the first step of glutathione synthesis) inhibition on the cytotoxicity of 2C-T-X and 25TX-NBOMe (EC_{50} and EC_{80}) drugs in differentiated SH-SY5Y cells. Cells were pre-exposed to L-buthionine sulfoximine (BSO, 100 µM) for 24 hours, and then co-incubated with the drugs for an additional 24 hours (50 µM BSO + drugs, in grey). Following the incubation period, the cytotoxicity was evaluated by the NR uptake assay. Results are presented as mean with 95 % confidence interval (CI) from a minimum of 4 independent experiments (3 replicates each). Statistical analysis were obtained using two-way ANOVA followed by Šídák's multiple comparisons test.

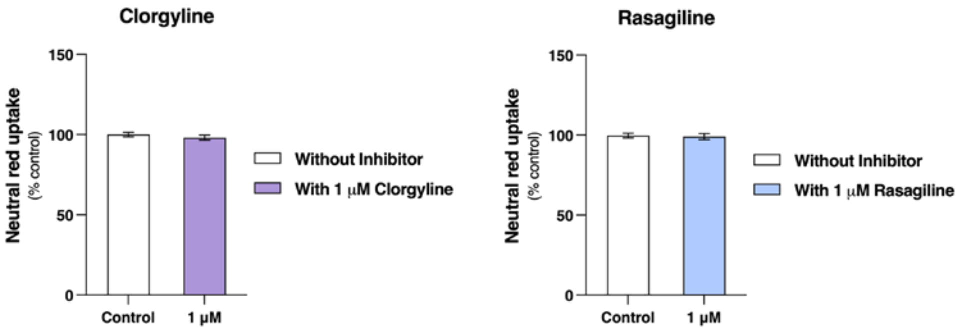

Supplementary Figure S5. Clorgyline and rasagiline (1 µM) cytotoxicity in differentiated SH-SY5Y cells evaluated by the NR uptake assay, after 24 hours of incubation. Results are presented as mean with 95 % confidence interval (CI) from at least 4 independent experiments (3 replicates each). Statistical comparisons were obtained using one-way ANOVA, followed by the Dunnett's multiple comparisons test.

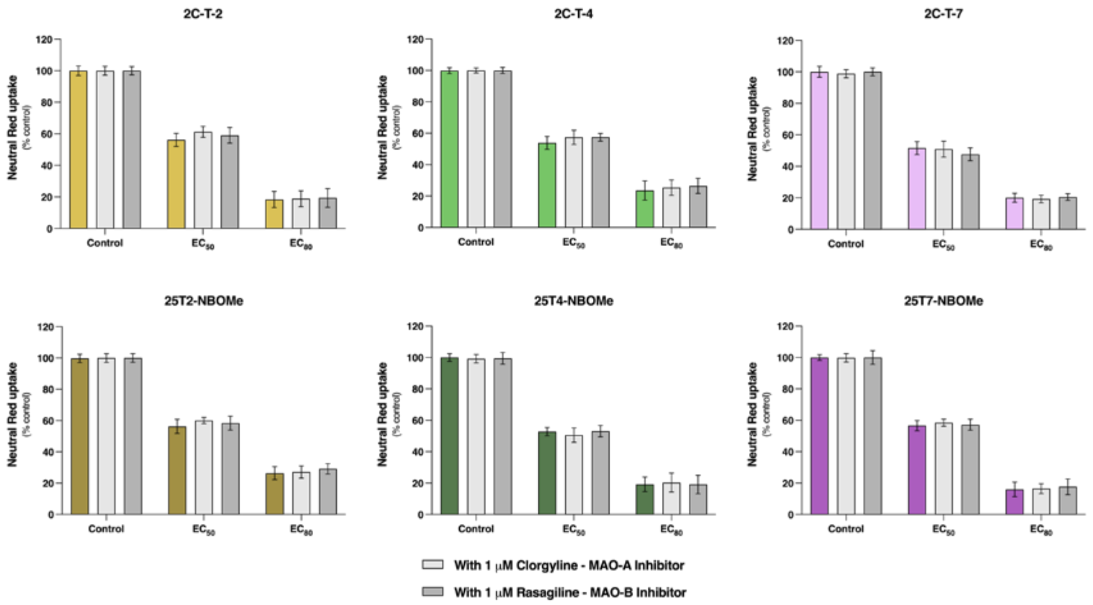

Supplementary Figure S6. Effect of monoamine oxidase (MAO) inhibition on the cytotoxicity of 2C-T-X (and 25TX-NBOMe (EC_{50} and EC_{80}) drugs in differentiated SH-SY5Y cells, following 24 hours of incubation with EC_{20} and EC_{50} of 2C-T-X and 25TX-NBOMe drugs, in the presence or absence of two pre-exposed (for 1 hour) MAO inhibitors: 1 μM clorgyline (MAO-A inhibitor, light gray) or 1 μM rasagiline (MAO-B inhibitor, dark grey). Following the incubation period, the cytotoxicity was evaluated by the NR uptake assay. Results are presented as mean with 95 % confidence interval (CI) from a minimum of 4 independent experiments (3 replicates each). Statistical analisys were obtained using two-way ANOVA followed by Tukey's multiple comparisons test.
